# Regulatory feedback cycle of the insulin‐degrading enzyme and the amyloid precursor protein intracellular domain: Implications for Alzheimer’s disease

**DOI:** 10.1111/acel.13264

**Published:** 2020-10-31

**Authors:** Anna A. Lauer, Janine Mett, Daniel Janitschke, Andrea Thiel, Christoph P. Stahlmann, Cornel M. Bachmann, Felix Ritzmann, Bianca Schrul, Ulrike C. Müller, Reuven Stein, Matthias Riemenschneider, Heike S. Grimm, Tobias Hartmann, Marcus O. W. Grimm

**Affiliations:** ^1^ Experimental Neurology Saarland University Homburg/Saar Germany; ^2^ Biosciences Zoology/Physiology‐Neurobiology Faculty NT‐Natural Science and Technology Saarland University Saarbrücken Germany; ^3^ Department of Internal Medicine V – Pulmonology Allergology, Respiratory Intensive Care Medicine Saarland University Hospital Homburg/Saar Germany; ^4^ Medical Biochemistry and Molecular Biology Center for Molecular Signaling (PZMS) Faculty of Medicine Saarland University Homburg/Saar Germany; ^5^ Department of Functional Genomics Institute of Pharmacy and Molecular Biotechnology Heidelberg University Germany; ^6^ Department of Neurology George S. Wise Faculty of Life Sciences Tel Aviv University Ramat Aviv Israel; ^7^ Department of Psychiatry and Psychotherapy Saarland University Hospital Homburg/Saar Germany; ^8^ Deutsches Institut für DemenzPrävention (DIDP) Saarland University Homburg/Saar Germany

**Keywords:** Alzheimer's disease, APP intracellular domain, Aβ homeostasis, Aβ‐degradation, insulin‐degrading enzyme

## Abstract

One of the major pathological hallmarks of Alzheimer´s disease (AD) is an accumulation of amyloid‐β (Aβ) in brain tissue leading to formation of toxic oligomers and senile plaques. Under physiological conditions, a tightly balanced equilibrium between Aβ‐production and ‐degradation is necessary to prevent pathological Aβ‐accumulation. Here, we investigate the molecular mechanism how insulin‐degrading enzyme (IDE), one of the major Aβ‐degrading enzymes, is regulated and how amyloid precursor protein (APP) processing and Aβ‐degradation is linked in a regulatory cycle to achieve this balance. In absence of Aβ‐production caused by APP or Presenilin deficiency, IDE‐mediated Aβ‐degradation was decreased, accompanied by a decreased IDE activity, protein level, and expression. Similar results were obtained in cells only expressing a truncated APP, lacking the APP intracellular domain (AICD) suggesting that AICD promotes *IDE* expression. In return, *APP* overexpression mediated an increased *IDE* expression, comparable results were obtained with cells overexpressing C50, a truncated APP representing AICD. Beside these genetic approaches, also AICD peptide incubation and pharmacological inhibition of the γ‐secretase preventing AICD production regulated *IDE* expression and promoter activity. By utilizing CRISPR/Cas9 APP and Presenilin knockout SH‐SY5Y cells results were confirmed in a second cell line in addition to mouse embryonic fibroblasts. In vivo, *IDE* expression was decreased in mouse brains devoid of APP or AICD, which was in line with a significant correlation of *APP* expression level and *IDE* expression in human *postmortem* AD brains. Our results show a tight link between Aβ‐production and Aβ‐degradation forming a regulatory cycle in which AICD promotes Aβ‐degradation via IDE and IDE itself limits its own production by degrading AICD.

## Graphical abstract

AICD‐mediated regulatory feedback cycle of IDE and APP processing.

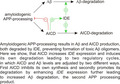

## 1 INTRODUCTION

Currently, more than 50 million people globally are estimated to suffer from dementia. Alzheimer’s disease (AD) is a progressive, irreversible neurodegenerative disease which is the most common cause of dementia in the elderly. The excessive accumulation and aggregation of the amyloid‐β (Aβ) peptide in brain tissue leading to the formation of extracellular senile plaques is considered to represent the initial pathological process of the disease characterized by synaptic loss and neuronal injury (Chen et al., 2017). Aβ peptides are products of the sequential amyloidogenic processing of the type I transmembrane amyloid precursor protein (APP), a member of a conserved protein family also including the APP‐like proteins 1 and 2 (APLP1 and APLP2), by β‐ and γ‐secretase (Figure S1). Beside the amyloidogenic APP processing pathway, APP can be cleaved in the predominant α‐ and γ‐secretase dependent non‐amyloidogenic cleavage cascade precluding the generation of Aβ peptides. In both APP processing pathways, cleavage of APP by γ‐secretase additionally leads to the release of the C‐terminal APP intracellular domain (AICD) into the cytosol. Due to multiple‐site cleavages by γ‐secretase, Aβ and AICD peptides can vary in length with the main products being Aβ38, Aβ40, Aβ42, and AICD C50, C53, C57, C59, respectively (Chen et al., 2017; Grimm et al., 2013).

Total cerebral Aβ level is not only determined by Aβ‐production, but also by Aβ‐clearance and degradation mechanisms, which have been reported to be impaired in the predominant late onset form of AD (Mawuenyega et al., 2010). These Aβ‐clearance mechanisms include among others the enzymatic elimination of Aβ peptides by proteases like insulin‐degrading enzyme (IDE) and neprilysin (NEP) (Nalivaeva & Turner, 2019). IDE is a zinc metallopeptidase most abundant in the cytosol, but also in several other subcellular compartments (Saido & Leissring, 2012) and represents one of the most important Aβ‐degrading enzymes in brain tissue. IDE deficient mice show increased cerebral accumulation of Aβ peptides while amyloid plaque formation is reduced in the brain tissue of mice with transgenic overexpression of IDE (Farris et al., 2003; Leissring et al., 2003; Miller et al., 2003).

Besides Aβ, AICD has also been demonstrated to be degraded by IDE in vitro and in vivo (Farris et al., 2003; Miller et al., 2003). AICD has been reported to be involved in the transcriptional regulation of several target genes including *APP*, *BACE1*, *NEP*, key enzymes of different lipid pathways and the mitochondrial master transcriptional coactivator *PGC*‐*1α* (Grimm et al., 2015; Pardossi‐Piquard et al., 2005; Robinson et al., 2014; von Rotz et al., 2004). The rapid cytosolic breakdown of AICD peptides by IDE and other enzymes might be precluded by binding to adaptor proteins like Fe65 enabling the translocation of AICD to the nuclear compartment (Kimberly et al., 2001). Within the nucleus, a trimeric protein complex consisting of AICD, Fe65, and the histone acetyltransferase Tip60 (AFT‐complex), which functions in transcriptional regulation, is formed (Cao & Sudhof, 2001; von Rotz et al., 2004).

In this study, we identified the Aβ‐degrading protease IDE as a target gene of AICD nuclear signaling. Hence, the two major Aβ‐degrading enzymes IDE and NEP are transcriptionally upregulated by AICD. This indicates the existence of a regulatory cycle in which proteolytic APP processing generates Aβ peptides and concurrently ensures their enzymatic degradation.

## 2 RESULTS

### 2.1 Total Aβ‐degradation is reduced in MEF cells devoid of PS1/2, APP/APLP2, and AICD

**Figure 1:**
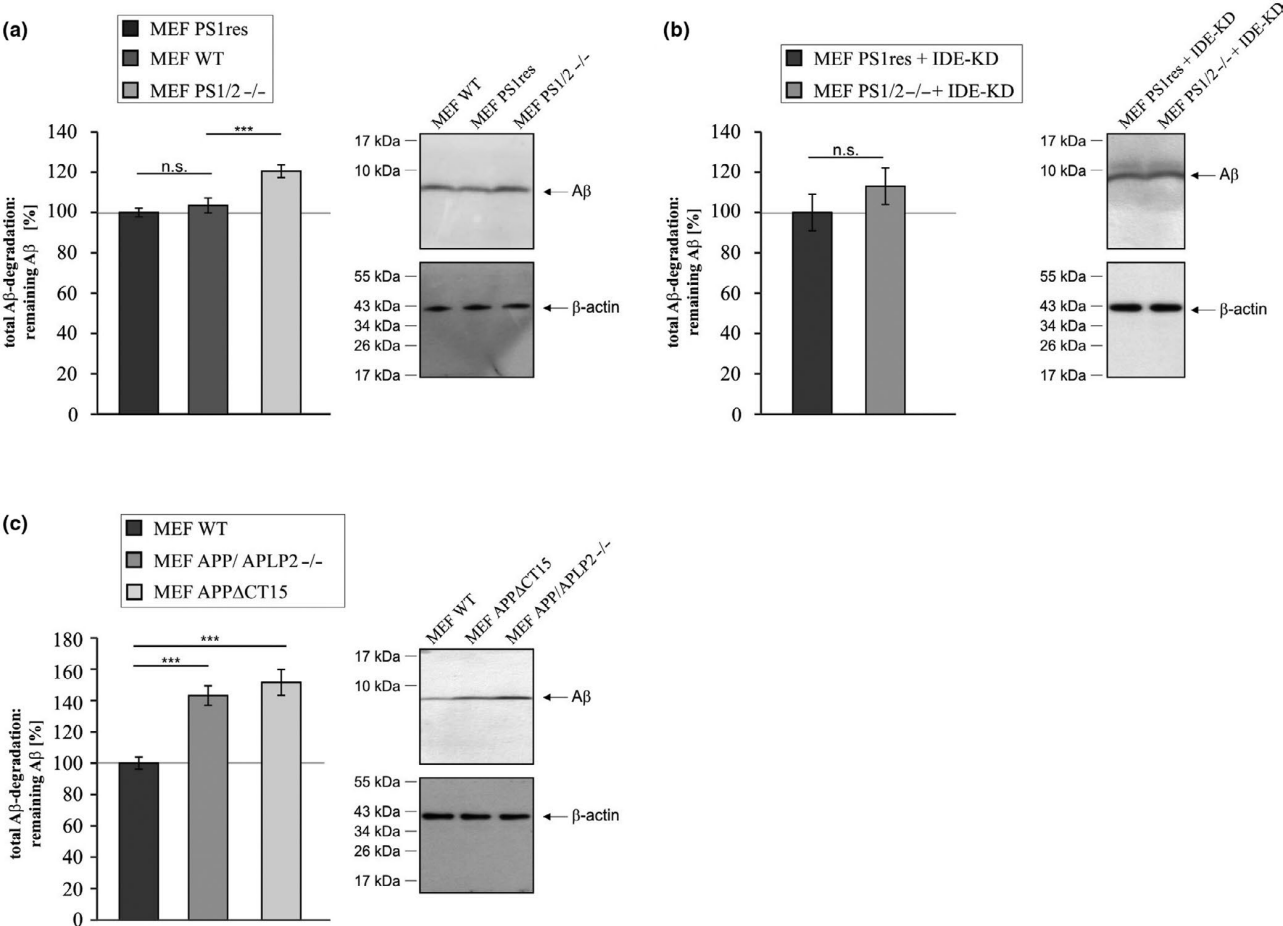
Aβ‐degradation. (a) Mouse embryonic fibroblasts devoid of PS1 and PS2 (MEF PS1/2−/−) and MEF WT cells compared to MEF PS1/2−/− retransfected with PS1 (MEF PS1res). (b) MEF PS1/2−/− transiently knocked‐known for insulin‐degrading enzyme (MEF PS1/2−/− + IDE‐KD) compared to MEF PS1res with a transient IDE knockdown (MEF PS1res + IDE‐KD). (c) Mouse embryonic fibroblasts devoid of the APP protein family (MEF APP/APLP2−/−) and mouse embryonic fibroblasts expressing truncated APP lacking a functional AICD domain (MEF APPΔCT15) compared to MEF WT. (a–c) Total Aβ‐degradation was determined by addition of human synthetic Aβ40 peptides to corresponding cell lysates. Remaining human synthetic Aβ40 peptides were determined by Western blot (WB) analysis with antibody W02 recognizing human but not endogenous murine Aβ peptides. Corresponding WBs are shown. No significant differences in β‐actin signals exist between the two compared cell lines (MEF WT: 109.4%, *p* = 0.640; MEF PS1/2−/−: 104.3%, *p* = 0.877; MEF PS1/2−/− + IDE‐KD: 103.5%, *p* = 0.441; MEF APP/APLP2−/−: 90.4%, *p* = 0.357; MEF APPΔCT15: 95.2%, *p* = 0.111). Statistical significance was calculated as described in Table S3. Error bars represent the standard error of the mean and significance was set at *p ≤ 0.05, **p ≤ 0.01 and ***p ≤ 0.001

In order to analyze the impact of the catalytically active subunit of the γ‐secretase complex, the presenilins (PS), on total intracellular Aβ‐degrading activity we used mouse embryonic fibroblasts (MEFs) devoid of PS1 and PS2 (MEF PS1/2−/−) and PS1 retransfected control cells (MEF PS1res) to avoid clonal heterogeneity (Figure S2A). Total intracellular Aβ‐degradation was measured by the addition of synthetic human Aβ40 peptides to the cell lysates for 1 h and subsequent quantification of the remaining, not degraded human Aβ40. No significant difference in Aβ‐degradation was observed between MEF wild type (MEF WT) and MEF PS1res (Figure 1a, Table 1). Considering the known AICD‐dependent transcriptional regulation of *NEP* (Grimm et al., 2015), total Aβ‐degradation was significantly reduced in MEF PS1/2−/− compared to MEF PS1res cells since remaining human Aβ peptides were significantly increased to 120.5% in PS1/2−/− cells (Figure 1a, Table 1). The magnitude of effect of PS1/2‐deficiency on total Aβ‐degradation was less pronounced after transient IDE knockdown (knockdown efficiency 56%, see Figure S2B) (remaining Aβ in MEF PS1/2−/− IDE knockdown: 113.0% ± 9.1%, *p* = 0.339) (Figure 1b) compared to the same experiment where IDE was not knocked down. This indicates that besides NEP IDE might also be affected by a lack of γ‐secretase activity. Besides APP more than 90 other substrates processed by the γ‐secretase complex have been identified (Wolfe, 2020). Therefore, we elucidated whether the effect of PS1/PS2‐deficiency on total Aβ‐degrading activity is depending on APP and its γ‐secretase dependent cleavage products Aβ and AICD. Aβ‐degradation was measured in MEF cells lacking full‐length APP and APLP2 (MEF APP/APLP2−/−) (cell line controlled in Figure S2C) or exclusively the APP C‐terminus (MEF APPΔCT15) (cell line controlled in Figure S2D). As *APLP1* expression is restricted to neurons (Thinakaran et al., 1995), MEF APP/APLP2−/− are devoid of the whole APP protein family. In contrast, MEF APPΔCT15 cells lack the last 15 C‐terminal amino acids (aa) of APP including the YENPTY motif required for nuclear targeting of AICD (Kimberly et al., 2001). As shown in Figure 1c, total Aβ‐degradation was significantly impaired in MEF APP/APLP2−/− as well as in MEF APPΔCT15 cells compared to MEF WT (remaining Aβ in MEF APP/APLP2−/−: 143.0% ± 6.3%, *p* ≤ 0.001; remaining Aβ in MEF APPΔCT15: 151.5% ± 8.2%, *p* ≤ 0.001) (Figure 1c). In presence of insulin, acting as a competitive inhibitor for IDE dependent Aβ degradation, remaining Aβ peptides were still significantly increased in MEF APP/APLP2−/− compared to MEF WT cells (Figure S4B), but the magnitude of effect between the MEF APP/APLP2−/− compared to MEF WT was less pronounced as in cells not treated with an IDE inhibitor (Figure S4A). A similar result was obtained in presence of the NEP inhibitor thiorphan (Figure S4C). Notably, no significant alterations between MEF WT and MEF APP/APLP2−/− in Aβ degradation were observed in presence of both inhibitors, insulin and thiorphan (Figure S4D).

**Table 1:** Overview of results shown in Figures 1, 2, 3, 4, 5, 6. Mean ± *SEM* and *p*‐value

				*p*‐Value
Figure 1	Total Aβ‐degradation: remaining Aβ			
	A	MEF PS1res (100%) vs. MEF WT	103.5% ± 3.7%	0.526
		MEF PS1res (100%) vs. MEF PS1/2−/−	120.5% ± 3.2%	0.000
	B	MEF PS1res + IDE‐KD (100%) vs. MEF PS1/2−/− + IDE‐KD	113.0% ± 9.1%	0.339
	C	MEF WT (100%) vs. MEF APP/APLP2−/−	143.0% ±6.3%	0.000
		MEF WT (100%) vs. MEF APPΔCT15	151.5% ±8.2%	0.000
Figure 2	IDE activity			
	A	MEF PS1res (100%) vs. MEF PS1/2−/−	88.5% ± 2.5%	0.019
	B	MEF WT (100%) vs. MEF APP/APLP2−/−	82.6% ± 1.7%	0.005
		MEF WT (100%) vs. MEF APPΔCT15	73.9% ± 4.9%	0.002
	IDE protein level			
	C	MEF PS1res (100%) vs. MEF PS1/2−/−	69.8% ± 3.8%	0.000
		MEF PS1res (100%) vs. PS1res + DAPT	68.7% ± 5.9%	0.000
	D	MEF WT (100%) vs. MEF APP/APLP2−/−	41.4% ± 4.2%	0.000
		MEF WT (100%) vs. MEF APPΔCT15	59.3% ± 10.4%	0.007
Figure 3	IDE gene expression			
	A	MEF PS1res (100%) vs. MEF PS1/2−/−	74.7% ± 7.2%	0.010
	B	MEF WT (100%) vs. MEF APP/APLP2−/−	76.6% ± 6.4%	0.000
		MEF WT (100%) vs. MEF APPΔCT15	37.9% ± 9.7%	0.001
	C	SH‐SY5Y WT (100%) vs. SH‐SY5Y PS1−/−	87.2% ± 5.7%	0.015
	D	SH‐SY5Y WT (100%) vs. SH‐SY5Y APP−/−	51.6% ± 3.3%	0.002
		SH‐SY5Y WT (100%) vs. SH‐SY5Y + APP^695^	169.9% ± 29.4%	0.005
	E	MEF APP/APLP2−/− (100%) vs. MEF APP/APLP2−/− + APP^695^	131.6% ± 6.9%	0.047
		MEF APP/APLP2−/− (100%) vs. MEF APP/APLP2−/− + APP^751^	134.0% ± 6.9%	0.022
		MEF APP/APLP2−/− (100%) vs. MEF APP/APLP2−/− + APP^770^	134.5% ± 4.4%	0.018
		MEF APP/APLP2−/− (100%) vs. MEF WT	186.6% ± 14.5%	0.000
Figure 4	IDE gene expression			
	A	MEF APPΔCT15 control (100%) vs. MEF APPΔCT15 + C50	131.4% ± 11.6%	0.005
		MEF APPΔCT15 control (100%) vs. MEF APPΔCT15 + AICD 48 h	139.6% ± 10.2%	0.000
		MEF APPΔCT15 control (100%) vs. MEF APPΔCT15 + AICD 9d	145.4% ± 12.3%	0.000
	B	SH‐SY5Y control (100%) vs. SH‐SY5Y + C50	147.8% ± 10.4%	0.002
	IDE protein level			
	C	MEF APPΔCT15 control (100%) vs. MEF APPΔCT15 + C50	135.9% ± 6.4%	0.004
		MEF APPΔCT15 control (100%) vs. MEF APPΔCT15 + AICD 48 h	128.4% ± 8.5%	0.027
	D	MEF PS1/2−/− control (100%) vs. MEF PS1/2−/− + AICD (48 h)	135.9% ± 2.3%	0.000
	Total Aβ‐degradation: remaining Aβ			
	E	MEF PS1res (100%) vs. MEF PS1/2−/−	120.5% ± 3.2%	0.000
		MEF PS1res (100%) vs. MEF PS1/2−/− + C50	104.0% ± 4.2%	0.675
Figure 5	IDE promoter activity			
	A	MEF WT (100%) vs. MEF APP/APLP2−/−	32.2% ± 2.3%	0.004
		MEF WT (100%) vs. MEF APPΔCT15	57.5% ± 2.4%	0.001
	B	MEF APPΔCT15 control (100%) vs. MEF APPΔCT15 + C50	125.7% ± 4.1%	0.003
Figure 6	IDE gene expression			
	A	Brain WT mice (100%) vs. brain APP−/− mice	86.9% ± 4.8%	0.014
		Brain WT mice (100%) vs. brain APPΔCT15+/− mice	91.7% ± 2.9%	0.007
	IDE protein level			
	B	Brain WT mice (100%) vs. brain APPΔCT15+/− mice	77.3% ± 4.9%	0.041
	Correlation *IDE*/*APP* gene expression			
	C	Cohort 1 (Braak stages 4–6)	*r* = 0.455	0.000
	D	Cohort 2 (Braak stages 1–3)	*r* = 0.261	0.033
Figure S4	Total Aβ‐degradation: remaining Aβ			
	A	MEF WT (100%) vs. MEF APP/APLP2−/−	143.0% ± 6.3%	0.000
	B	MEF WT (100%) vs. MEF APP/APLP2−/−	114.4% ± 3.1%	0.006
	C	MEF WT (100%) vs. MEF APP/APLP2−/−	117.3% ± 4.3%	0.035
	D	MEF WT (100%) vs. MEF APP/APLP2−/−	104.5% ± 7.7%	0.686

These results indicate that the PS‐dependent APP cleavage product AICD might also be involved in the regulation of IDE besides the reported influence of AICD on NEP (Grimm et al., 2015).

### 2.2 IDE enzyme activity and protein level are reduced in MEF cells devoid of PS1/2, APP/APLP2, and AICD

**Figure 2:**
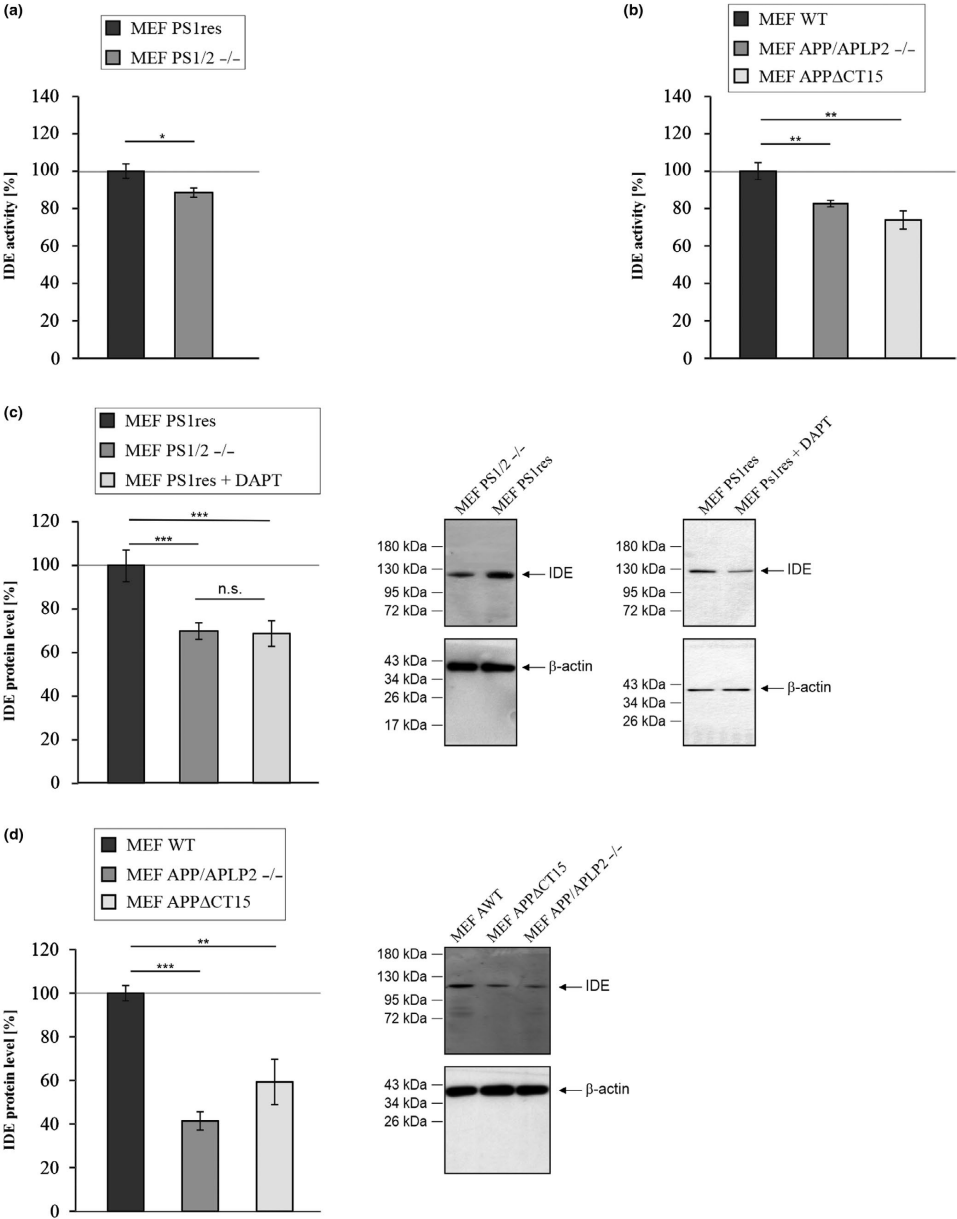
Determination of IDE enzyme activity and IDE protein level in mouse embryonic fibroblasts devoid of PS1/2 (MEF PS1/2−/−), APP/APLP2 (MEF APP/APLP2−/−) or AICD (MEF APPΔCT15). (a) IDE enzyme activity in MEF PS1/2−/− compared to MEF PS1/2−/− retransfected with PS1 (MEF PS1res). (b) Reduced IDE enzyme activity in mouse embryonic fibroblasts devoid of the APP family (MEF APP/APLP2−/−) or devoid of a functional AICD domain (MEF APPΔCT15) compared to wildtype cells (MEF WT). (c) IDE protein level determined by WB analysis in MEF PS1/2−/− cells or MEF PS1res cells incubated with the γ‐secretase inhibitor DAPT compared to MEF PS1res. (d) IDE protein level in MEF APP/ALPL2−/− and MEF APPΔCT15 cells compared to MEF WT. Corresponding WBs are shown. No significant differences in β‐actin signals exist between the two compared cell lines (MEF PS1/2−/−: 106.8%, *p* = 0.099; MEF PS1/2−/− + DAPT: 106.1%, *p* = 0.761; MEF APP/APLP2−/−: 93.2%, *p* = 0.769; MEF APPΔCT15: 112.7%, *p* = 0.518). Statistical significance was calculated as described in Table S3. Error bars represent the standard error of the mean and significance was set at *p ≤ 0.05, **p ≤ 0.01 and ***p ≤ 0.001

In order to analyze whether the PS/APP/AICD‐dependent effects on total Aβ‐degradation are partially based on an altered IDE activity, we measured IDE enzyme activity in MEF PS1/2−/−, MEF APP/APLP2−/− and in MEF APPΔCT15 compared to the corresponding control cell lines. The enzymatic activity of the protease was significantly reduced in PS1/2‐deficient cells (Figure 2a, Table 1) as well as in APP/APLP2‐deficient cells and in cells devoid of AICD (Figure 2b, Table 1). As shown in Figure 2c,d these effects are based on a significant reduction of IDE protein level. In MEF PS1/2−/− cells lacking the catalytic subunit of the γ‐secretase complex, IDE protein content was decreased to 69.8% compared to MEF PS1res (Figure 2c, Table 1). Importantly, a similar effect was also observed by inhibition of γ‐secretase activity in the PS1 retransfected control cells demonstrating IDE protein level to be strongly dependent on γ‐secretase activity (MEF PS1res + DAPT: 68.7% ± 5.9%, *p* ≤ 0.001) (Figure 2c, Table 1). Similarly, IDE protein level was found to be significantly decreased in MEF cells lacking the APP family (MEF APP/APLP2−/−) or AICD (MEF APPΔCT15) (Figure 2d, Table 1). These results further support a mechanism in which IDE might be regulated in an AICD‐dependent manner.

### 2.3 Influence of PS, APP, and AICD on IDE gene expression in MEF and SH‐SY5Y cells

**Figure 3:**
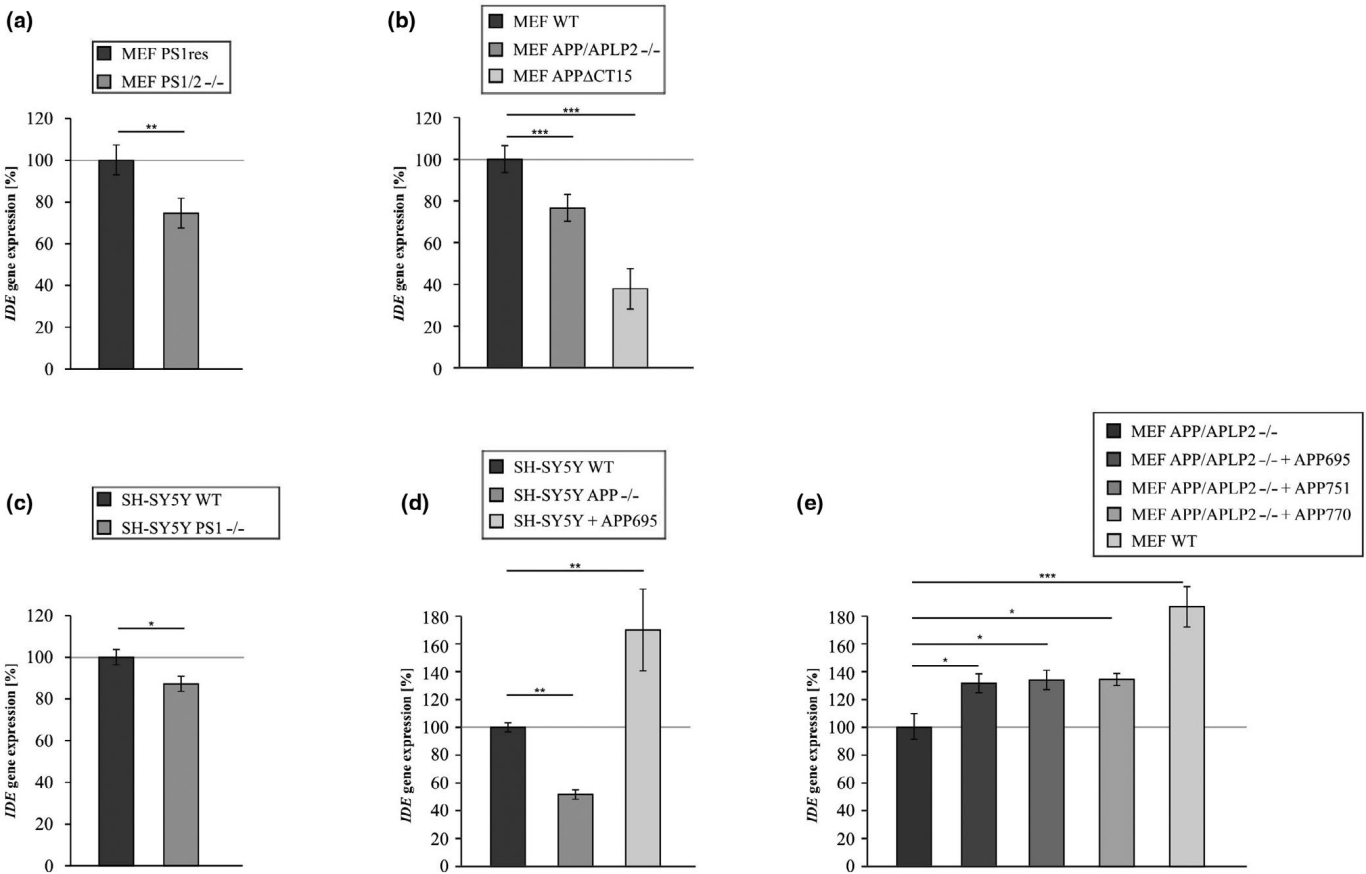
*IDE* gene expression determined by RT‐PCR in different cell lines devoid of the catalytically active components of the γ‐secretase complex, the APP family or AICD and in APP overexpressing cell lines. (a) *IDE* gene expression in mouse embryonic fibroblasts devoid of PS1 and PS2 (MEF PS1/2−/−) compared to wildtype cells (MEF WT). (b) Impaired *IDE* gene transcription in mouse embryonic fibroblasts devoid of the APP family (MEF APP/APLP2−/−) or devoid of a functional AICD domain (MEF APPΔCT15). (c) Reduced *IDE* gene expression in the human neuroblastoma cell line SH‐SY5Y knocked out for PS1 (SH‐SY5Y PS1−/−) compared to wildtype cells (SH‐SY5Y WT). PS1 knockout was generated using CRISPR‐Cas9. (d) *IDE* gene expression in SH‐SY5Y cells knocked out for APP using CRISPR‐Cas9 (SH‐SY5Y APP−/−) and SH‐SY5Y cells stably overexpressing APP695 (SH‐SY5Y APP695). (e) *IDE* gene transcription in MEF APP/APLP2−/− cells retransfected with the main APP isoforms APP695 (MEF APP/APLP2−/− + APP695), APP751 (MEF APP/APLP2−/− + APP751) or APP770 (MEF APP/APLP2−/− + APP770) and in wildtype cells (MEF WT) compared to MEF APP/APLP2−/−. Statistical significance was calculated as described in Table S3. Error bars represent the standard error of the mean and significance was set at *p ≤ 0.05, **p ≤ 0.01 and ***p ≤ 0.001

AICD has been reported to translocate to the nucleus and to be involved in the regulation of several target genes (Grimm et al., 2015; Pardossi‐Piquard et al., 2005; Robinson et al., 2014; von Rotz et al., 2004). To examine whether the reduction of IDE protein level and enzyme activity in cells devoid of PS, APP, or AICD is caused by a decreased *IDE* gene expression in absence of AICD, we performed real‐time PCR (RT‐PCR) analyses of the corresponding cell lines. In line with the AICD‐dependent regulation of *IDE* by AICD, *IDE* gene expression was found to be significantly reduced in the MEF cells lacking PS1/2, APP/APLP2, or the APP C‐terminus (MEF APPΔCT15) (Figure 3a+b, Table 1). Next, we tested whether the AICD‐dependent transcriptional regulation of *IDE* is restricted to MEF cells. Considering the important Aβ‐degrading function of IDE in human brain, we decided to use the human neuroblastoma cell line SH‐SY5Y as a second cellular model. In line with the findings obtained in the different MEF cell lines, *IDE* gene expression was significantly reduced in SH‐SY5Y cells devoid of PS1 (SH‐SY5Y PS1−/−) (Figure 3c, Table 1, cell line controlled in Figure S2H) or APP (SH‐SY5Y APP−/−) (Figure 3d, Table 1, cell line controlled in Figure S2G). In accordance on the other hand overexpression of APP695, the most common APP isoform in neuronal cells resulted in a significantly increased *IDE* gene expression in SH‐SY5Y cells (SH‐SY5Y + APP695) (Figure 3d, Table 1, cell line controlled in Figure S2E). As the nuclear localization and gene regulatory activity is discussed to be restricted to AICD derived from APP695 (Belyaev et al., 2010), we decided to analyze the impact of different APP isoforms on *IDE* gene expression. Therefore, MEF APP/APLP2−/− cells were transiently retransfected with plasmids encoding for APP695, APP751, and APP770, the three major splice isoforms of APP. The levels of *APP* expression were significantly increased in all isoform‐expressing cells compared to the mock‐transfected control cells (Figure S2F). All three APP isoforms upregulated *IDE* gene expression to a similar extent compared to mock‐transfected MEF APP/APLP2−/− control cells (Figure 3e, Table 1), but did not reach the level of MEF WT cells.

### 2.4 Impact of AICD on *IDE* gene expression and IDE protein level

**Figure 4:**
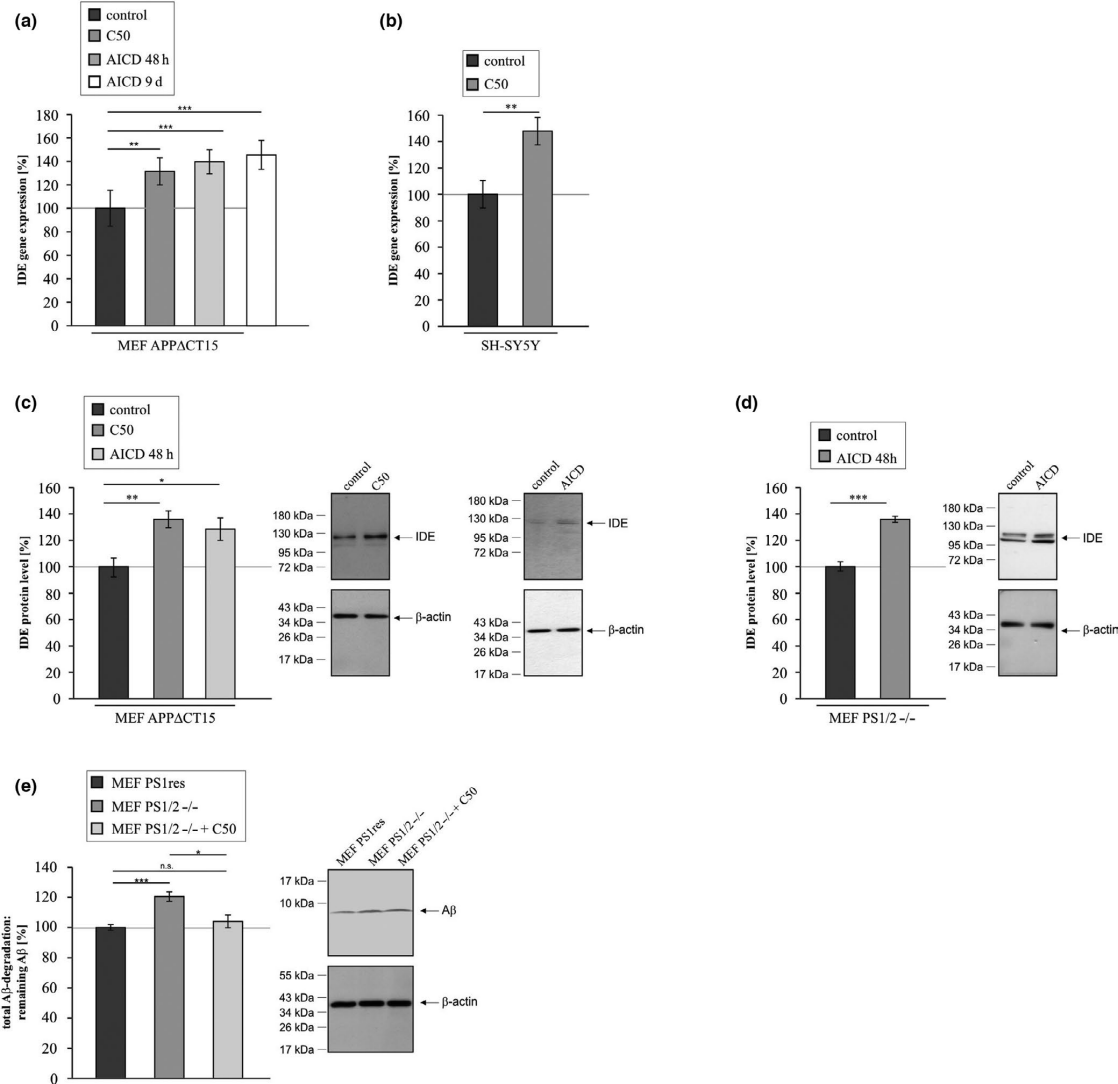
Analysis of *IDE* gene expression and IDE protein level in presence of AICD. (a) *IDE* gene expression determined by RT‐PCR in MEF APPΔCT15 transfected with a plasmid encoding for the last 50 aa of the APP C‐terminus (C50) or in MEF APPΔCT15 short‐ and long‐term incubated with AICD peptides (AICD). (b) Increased *IDE* gene expression in SH‐SY5Y cells stably expressing C50 compared to mock‐transfected control cells. (c) Elevated IDE protein level in MEF APPΔCT15 cells transfected with C50 or incubated for 48 h with AICD peptides. (d) Increased IDE protein level in MEF PS1/2−/− cells after incubation with AICD peptides for 48 h. (e) Aβ degradation in MEF PS1res, MEF PS1/2−/− and MEF PS1/2−/− cells transfected with C50. Corresponding WBs are shown. No significant differences in β‐actin signals exist between the two compared cell lines (MEF APPΔCT15 + C50: 96.8%, *p* = 0.771; MEF APPΔCT15 + AICD: 104.6%, *p* = 0.664; MEF PS1/2−/− + AICD: 109.8, *p* = 0.599; MEF PS1/2−/−: 104.3%, *p* = 0.877; MEF PS1/2−/− + C50: 101.5%, *p* = 0.892). Statistical significance was calculated as described in Table S3. Error bars represent the standard error of the mean and significance was set at * *p* ≤ 0.05, ** *p* ≤ 0.01 and *** *p* ≤ 0.001

To further strengthen the importance of AICD in the regulation of IDE we transiently transfected MEF APPΔCT15 cells, lacking a functional AICD domain, with an AICD‐expressing plasmid corresponding to the last 50 aa of the APP C‐terminus (C50) (cell line characterized in Figure S2I). APPΔCT15 cells expressing C50 showed a significant increase of *IDE* gene expression to 131.4% compared to cells lacking AICD (MEF APPΔCT15) (Figure 4a, Table 1). MEF APPΔCT15 short‐ and long‐term incubation with AICD peptides also revealed a significant increase in *IDE* gene expression to 139.6% and 145.4%, respectively (Figure 4a, Table 1). Taking into consideration that both incubation times showed comparable effects and in order to save AICD, only the short‐term incubation was utilized in further experiments. Similarly, SH‐SY5Y cells stably transfected with C50 (cell line characterized in Figure S2J), significantly increased *IDE* gene expression to 147.8% compared to mock‐transfected SH‐SY5Y control cells (Figure 4b, Table 1). In line with the observed elevation of *IDE* gene expression by addition of AICD peptides to cultured MEF APPΔCT15 cells or transfection with C50, the IDE protein level was significantly increased to 128.4% in presence of AICD peptides for 48 h and to 135.9% after transient transfection with C50 (Figure 4c, Table 1). A significant elevation in IDE protein level to 135.9% was also found for PS‐deficient MEF incubated with AICD peptides for 48 h (Figure 4d, Table 1). In agreement with the observed increase in IDE protein level after transient transfection with C50 or incubation with AICD peptides, the impaired Aβ degradation found for MEF PS1/2−/− (Figure 1a) could be rescued by transient transfection with C50. A transient transfection of PS1/2−/− with C50 was able to rescue the Aβ degradation, so that no significant difference between MEF PS1res and MEF PS1/2−/− C50 transfected cells could be observed. Notably, compared to MEF PS1/2−/− remaining Aβ peptides were significantly reduced in PS‐deficient MEF transfected with C50 (Figure 4e, Table 1).

### 2.5 The effect of a functional AICD domain on IDE promoter activity

**Figure 5:**
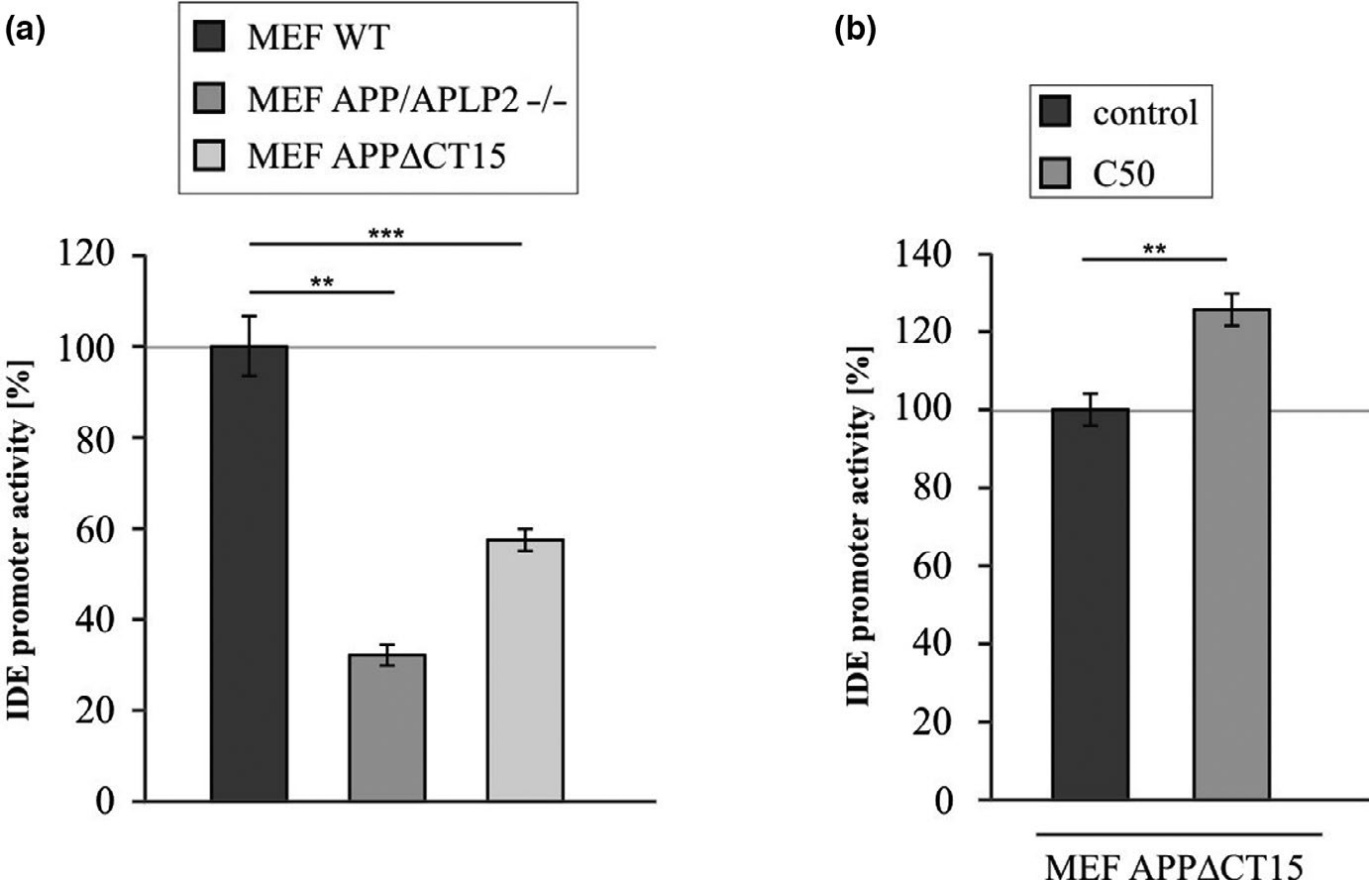
IDE promoter activity. (a) Reduced IDE promoter activity in cells lacking APP/APLP2 (MEF APP/APLP2−/−) or lacking a functional AICD domain (MEF APPΔCT15) compared to wildtype cells (MEF WT). (b) Increased IDE promoter activity in MEF APPΔCT15 cells transfected with C50. Cells were transiently transfected with the dual reporter system vector pEZX‐PG04‐IDE‐Gluc and *Gaussia* luciferase (GLuc) activity was measured with a fluorometric‐based assay. Statistical significance was calculated as described in Table S3. Error bars represent the standard error of the mean and significance was set at *p ≤ 0.05, **p ≤ 0.01 and ***p ≤ 0.001

Next, we analyzed whether IDE promoter activity is affected in cells lacking the APP protein family or a functional AICD domain. Therefore, cells were transiently transfected with the dual reporter system vector pEZX‐PG04‐IDE‐Gluc. The *Gaussia* luciferase gene (GLuc) acts as a reporter gene as its expression is regulated by the IDE promoter region. Luciferase activity and thus IDE promoter activity was significantly reduced in both MEF APP/APLP2−/− and MEF APPΔCT15 compared to MEF WT (Figure 5a, Table 1), indicating that AICD regulates the promoter region of the IDE coding sequence. Consistent with the other C50 rescue experiments, MEF APPΔCT15 transfected with C50 showed a significant increase in IDE promoter activity to 125.7% (Figure 5b, Table 1).

### 2.6 In vivo relevance of AICD‐dependent *IDE* gene expression

**Figure 6:**
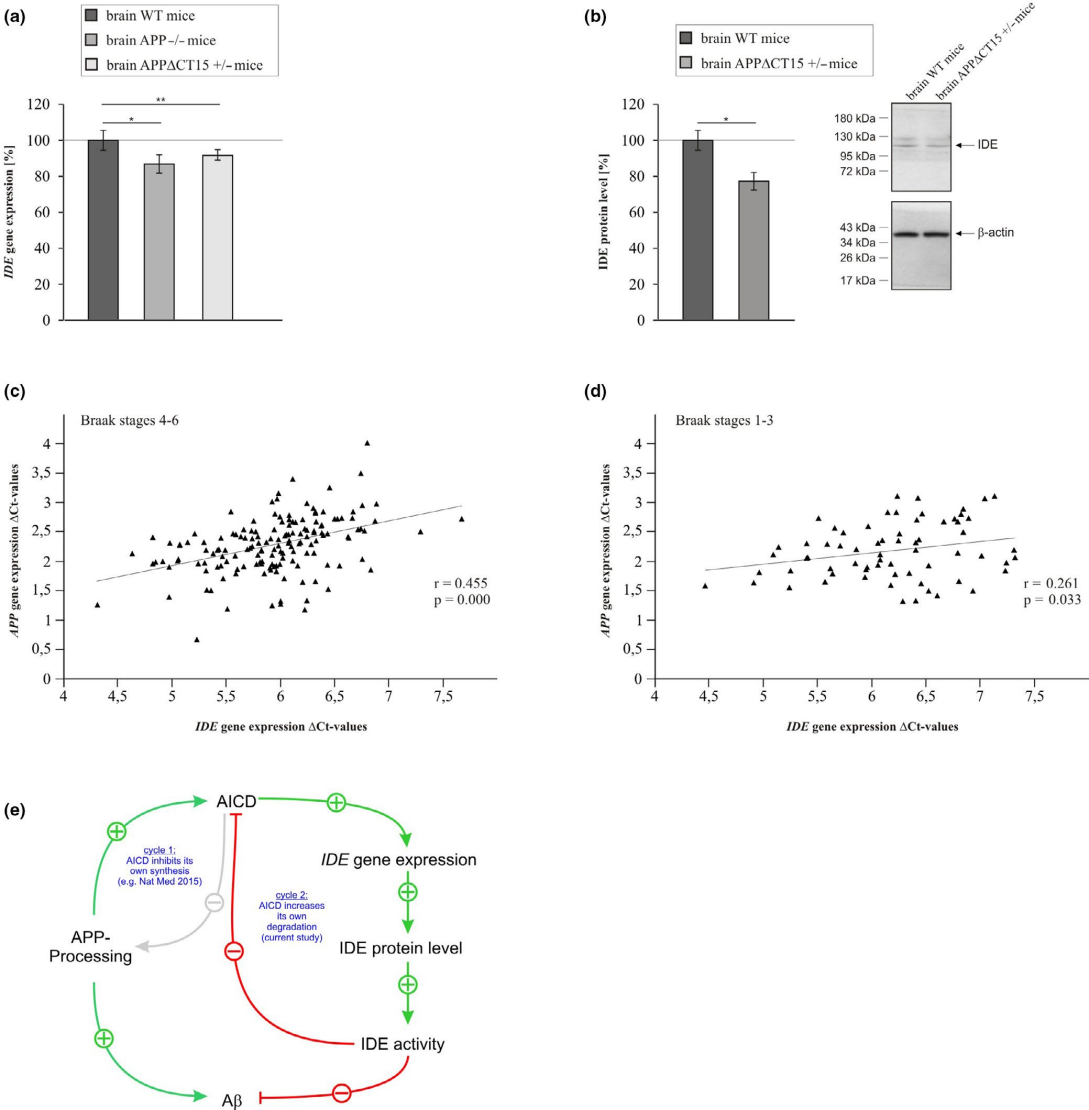
(a) *IDE* gene expression in brain homogenates of APP‐deficient mice (APP−/−) and of mice expressing truncated APP lacking the last 15 aa of the APP C‐terminus (APPΔCT15) compared to wildtype mice. (b) IDE protein level in brain homogenates of mice expressing truncated APP lacking the last 15 aa of the APP C‐terminus (APPΔCT15+/−). Corresponding WBs are shown. No significant differences in β‐actin signals exist between the two compared cell lines (APPΔCT15+/− mice: 105.5%, *p* = 0.406). Statistical significance was calculated as described in Table S3. Error bars represent the standard error of the mean and significance was set at *p ≤ 0.05, **p ≤ 0.01 and ***p ≤ 0.001. (c) Correlation of *APP*/*IDE* gene expression in human *postmortem* brains of 156 patients diagnosed with Braak stages 4–6. (d) Correlation of *APP*/*IDE* gene expression in human *postmortem* brains of 67 patients diagnosed with Braak stages 1–3. Statistical significance was calculated as described in Table S3. (e) Schematic overview of the proposed feedback cycles for AICD‐dependent IDE regulation and AICD‐dependent APP processing

*IDE* gene expression was monitored in APP knockout mice (APP−/−) and in heterozygous mice expressing the truncated APP lacking the last 15 aa of the C‐terminus (APPΔCT15+/−), to validate our findings in vivo. Brain homogenates of APP−/− mice showed a significant reduction in *IDE* gene expression (Figure 6a, Table 1). Similarly, *IDE* gene expression was significantly reduced in brain homogenates of APPΔCT15 expressing heterozygous transgenic mice (Figure 6a, Table 1). IDE protein level was also found to be significantly reduced in brain homogenates of APPΔCT15+/− mice (Figure 6b, Table 1).

To further investigate the in vivo relevance of an AICD‐dependent upregulation of *IDE* gene expression, we analyzed *IDE* as well as *APP* gene transcription in human *postmortem* brains of 156 AD affected individuals. *APP* gene expression positively correlated with *IDE* gene expression (*r* = 0.455) in patients with Braak stages 4–6 (Figure 6c), representing the later stages in AD. Notably, this correlation was highly significant (*p* ≤ 0.001), suggesting that our findings are not limited to cell culture or in vitro experiments. Also for patients with Braak stages 1–3 (67 patients) we found a positive significant correlation of *IDE* gene expression with *APP* gene expression (Figure 6d). The combination of both cohorts (Braak stages 1–6) revealed a significant positive correlation for *IDE* and *APP* gene expression (Figure S5A). In accordance to the positive correlation of *IDE* with *APP* gene expression we also found a significant positive correlation for the protein level of IDE with APP (Figure S5B) for samples (Braak 1–6) where enough amount of protein to perform Western blots were available.

No significant alterations in *IDE* and *APP* gene expression were observed for Braak stages 2–6 compared to Braak stage 1, representing early AD (Figure S5C). Similarly, amyloid burden did not influence *IDE* and *APP* gene expression (Figure S5D). The positive correlation between *IDE* and *APP* gene expression is not dependent on the gender as we obtained a significant positive correlation for both, women and men (Figure S5F). The ApoE status of the patients had no impact on *APP* and *IDE* gene expression (Figure S5E). Additionally, no significant correlations were obtained for age and *postmortem* delay (Figure S5G,H).

## 3 DISCUSSION

Extracellular senile plaques composed of aggregated Aβ peptides are one of the main pathological hallmarks of AD, however, oligomeric forms of Aβ seem to be the primary toxic species causing synaptic damage and neurodegeneration (Lambert et al., 1998; Umeda et al., 2011) and levels of soluble Aβ strongly correlate with markers of AD severity (McLean et al., 1999). Soluble extracellular Aβ peptides in the brain can be removed by efflux into the blood via the blood‐brain barrier (Tarasoff‐Conway et al., 2015) or can be degraded among others by IDE or NEP, which play also an important role in intracellular Aβ‐degradation (Iwata et al., 2001; Stargardt et al., 2013). NEP levels have been found to be reduced in hippocampus, temporal gyrus, and cortex of human *postmortem* AD brains (Grimm et al., 2013). However, there are still controversies in regard to the expression and activity of IDE in AD brains, showing reduced (Stargardt et al., 2013; Zhao et al., 2007), unchanged (Miners et al., 2010; Wang et al., 2010) or increased IDE activity (Miners et al., 2009; Morelli et al., 2004). Recently, we and others could show an AICD‐dependent regulation of NEP increasing its gene expression, protein level, and activity (Belyaev et al., 2009, 2010; Grimm et al., 2015; Pardossi‐Piquard et al., 2005; Xu et al., 2011). In our previous study, we could show that an AICD‐dependent change in Aβ‐degradation could only be partially rescued by utilizing thiorphan, a specific inhibitor of NEP. These results suggest that beside NEP another Aβ‐degrading protease might be also regulated by AICD (Grimm et al., 2015), which is in line with our recent finding that thiorphan could only partially attenuate the difference between MEF WT and MEF APP/APLP2−/− cells in respect to Aβ degradation.

In the present study, we identified *IDE* as a further target gene of AICD using both cells devoid of AICD or AICD generation and AICD overexpressing cells. We found *IDE* gene expression to be consistently downregulated in cells with impaired AICD generation (MEF PS1/2−/−; SH‐SY5Y PS1−/−), devoid of APP or the APP protein family (MEF APP/APLP2−/−, SH‐SY5Y APP−/−) or lacking a functional AICD domain (MEF APPΔCT15). mRNA levels of *IDE* were significantly downregulated in mouse embryonic fibroblasts knocked out for both catalytically active subunits of the γ‐secretase complex, PS1 and PS2. In line, *IDE* gene expression was also significantly reduced in human neuroblastoma PS1 knockout cells. Likely caused by the remaining expression of PS2 in this cell line, the observed effect strength on *IDE* mRNA level was not as pronounced as for PS1/2 lacking MEF cells. Due to the high number of substrates that can be cleaved by the γ‐secretase complex (Wolfe, 2020), we verified our findings in cells lacking APP and thus AICD. *IDE* gene expression was significantly reduced in SH‐SY5Y cells devoid of APP and in mouse embryonic fibroblasts lacking the APP family. Mouse embryonic fibroblasts expressing a truncated APP construct lacking the last 15 aa of the C‐terminus also showed a significant reduction in *IDE* mRNA level, further indicating an AICD‐dependent regulation of IDE. Importantly, the last 15 aa include the YENPTY motif known to interact with the adaptor protein FE65, increasing the stability of AICD (Kimberly et al., 2001) and enabling the transport of AICD to the nucleus where it associates with Tip60 leading to the formation of the AFT‐complex (Goodger et al., 2009; von Rotz et al., 2004).

In line with reduced *IDE* gene expression obtained for AICD deficient cells, we observed elevated *IDE* gene expression in cells overexpressing APP or the AICD encoding fragment C50 and in cells incubated with AICD peptides. *IDE* mRNA levels were significantly elevated in human neuroblastoma cells stably expressing APP695. Moreover, we observed no differences with respect to the expressed main APP isoforms, neuronal APP695 and non‐neuronal APP751 and APP770. APP695, APP751 and APP770 expressed in mouse embryonic fibroblasts devoid of the APP family, showed a nearly identical increase in *IDE* gene expression compared to APP/APLP2 knockout cells. However, the *IDE* gene expression level did not reach the level of WT fibroblasts, which showed an even stronger increase in *IDE* mRNA level. This might be caused by the endogenous expression of *APLP2* in WT fibroblasts, resulting in the γ‐secretase derived fragment of APLP2 (ALID2). It cannot be excluded that ALID2 might also be involved in the regulation of *IDE* gene expression as it has been reported that ALID2 influences the expression of the Aβ‐degrading enzyme NEP. Fibroblasts lacking APLP2 revealed reduced *NEP* expression and activity and NEP activity could be restored by retransfection with APLP2 (Pardossi‐Piquard et al., 2005). Beside ALID2 it has been shown that the γ‐secretase cleavage product of APLP1 (ALID1) increases NEP activity (Pardossi‐Piquard et al., 2005). The impact of ALID1 and ALID2 on *IDE* gene transcription has to be addressed in further studies. In contrast to our finding that all APP isoforms affected *IDE* mRNA levels, Nalivaeva et al. (2016) reported increased *IDE* gene expression for APP751 and APP770 overexpressing cells but not for cells overexpressing APP695. These divergent findings might be caused by the level of *APP* overexpression or the analyzed cell line and the impact of different APP isoforms on *IDE* gene transcription could be addressed in siRNA experiments silencing only one APP splice isoform.

Further illustrating an AICD‐dependent regulation of IDE we found *IDE* gene expression to be significantly increased in SH‐SY5Y cells stably expressing C50, encoding the AICD fragment of APP. Similarly, fibroblasts lacking a functional AICD domain significantly increased *IDE* gene expression when transfected with the C50 plasmid or incubated with AICD peptides. In line, C50 expression or AICD incubation in MEF APPΔCT15 and PS‐deficient cells revealed significantly elevated IDE protein level. Furthermore, the rescue of a functional AICD domain resulted in Aβ degradation similar to PS1res cells (see Figure 4e).

*Vice versa*, the IDE protein level was reduced in cells lacking the APP family, a functional AICD domain or in cells with impaired AICD production. Importantly, no statistical difference was observed in the protein levels of IDE in cells devoid of PS1/2 or WT fibroblasts incubated with the γ‐secretase inhibitor DAPT. Also, fibroblasts lacking the APP family or lacking a functional AICD domain consistently showed reduced IDE protein levels, resulting in a reduced IDE activity and impaired Aβ‐degradation as expected by the described AICD‐dependent regulation of *IDE* gene expression. A possible direct impact of AICD on IDE promoter activity could be proposed by our finding that fibroblasts lacking the APP family or a functional AICD domain showed reduced IDE promoter activity whereas MEF APPΔCT15 expressing C50 revealed significantly increased IDE promoter activity. This direct influence of AICD on IDE promoter activity might have an additional impact on the also discussed effect of the histone deacetylases HDAC1 and HDAC3 on the IDE promoter (Nalivaeva et al., 2016) found by the APP751 and APP770 isoforms.

Taken into consideration that we have shown in a previous study that AICD upregulates the expression of the peroxisome proliferator‐activated receptor γ coactivator‐1α (PGC‐1α) (Robinson et al., 2014) resulting in PPARγ activation, one might hypothesize that the AICD‐dependent upregulation of IDE found in the present study could be mediated by the PPARγ pathway. Interestingly, Du et al. (2009) reported that PPARγ plays an important role in regulating *IDE* expression in rat primary neurons through binding to a functional peroxisome proliferator‐response element (PPRE) in the IDE promoter, promoting *IDE* gene transcription. In vivo, the PPARγ activator rosiglitazone increased the expression level of *IDE* and decreased Aβ levels in a mixed mouse model of AD and type 2 diabetes and alleviated the spatial learning and recognition impairments in these mice (Li et al., 2018). Additionally, inhibition of PPARγ by injecting the PPARγ antagonist GW9662 in the fourth ventricle of APP/PS1 transgenic mice markedly decreased cerebellar levels of IDE and significantly induced Aβ levels (Du et al., 2009). In line with our hypothesis that PGC‐1α might be involved in the regulation of *IDE* gene transcription, Leal et al. (2013) reported a significant increase in cytosolic and mitochondrial levels of IDE in cells transfected with PGC‐1α. Besides increasing PPARγ transcriptional activity, PGC‐1α induces nuclear respiratory factor 1 (*NRF*‐*1*) overexpression, which has been found to bind to the IDE promoter region in vivo (Leal et al., 2013). Moreover, a strong positive correlation between PGC‐1α or NRF‐1 and the long mitochondrial IDE isoform was found in non‐demented brains, whereas this correlation was weaker in AD brains (Leal et al., 2013). The postulated AICD/PGC‐1α/PPARγ involvement in *IDE* transcriptional regulation might also play a role in the transcriptional regulation of *NEP* as it has been shown, that activation of the nuclear retinoid X receptor (RXR), the heterodimeric partner of PPARγ, upregulates not only IDE but also the Aβ degrading enzyme NEP (Nalivaeva et al., 2016). In the present study, we found some indications that the AICD/PGC‐1α/PPARγ pathway might indeed be involved in the regulation of IDE. *PGC‐1α* gene expression is significantly downregulated in SH‐SY5Y WT cells incubated with a γ‐secretase inhibitor and thus devoid of AICD generation (Figure S6A) which is in line with our previous finding that *PGC‐1α* mRNA level as well as protein level are decreased in PS‐deficient cells (Robinson et al., 2014). Additionally, we found that the magnitude of effect on *IDE* mRNA level between MEF WT and MEF APPΔCT15 cells in presence of a PPARγ inhibitor is significantly less pronounced than without inhibitor (Figure S6C) indicating the involvement of the PPARγ pathway in the regulation of IDE. Similarly, the effect strength on *IDE* gene expression was less pronounced in presence of a PGC‐1α‐inhibitor (Figure S6B). Although our data are in line with the models discussed in literature, further studies have to clarify the involvement of the PGC‐1α/PPARγ pathway in the regulation of IDE.

The in vivo relevance of our findings was assessed in APP knockout mouse brains and brains of heterozygous mice expressing a construct lacking a functional AICD domain. Brain homogenates of these mouse models revealed reduced *IDE* mRNA levels. Furthermore, we found a strong positive correlation of *APP* mRNA levels with *IDE* mRNA levels in *postmortem* brains of 223 patients in two cohorts (Braak stages 1–3 and Braak stages 4–6). Also, the IDE protein level correlated with the APP protein level in patients with Braak stages 1–6.

However, although the data of human *postmortem* brain tissue is in line with the other experimental data, it has to be emphasized that this data has to be interpreted carefully. The average *postmortem* time of 06:07 hours and the short half‐life of AICD make it impossible to directly correlate AICD levels with *IDE* expression (Kimberly et al., 2001). Instead, APP protein or RNA levels were analyzed and correlated with IDE making this approach more indirect.

In summary, we propose a feedback cycle for the AICD‐dependent regulation of IDE, in which AICD increases its own degradation as IDE has been also found to degrade AICD peptides (Edbauer et al., 2002). AICD upregulates *IDE* gene expression, either direct or by the above‐discussed involvement of the PGC‐1α/PPARγ pathway leading to increased IDE protein level and activity (Figure 6e). The increased IDE activity, in return, results in elevated degradation of Aβ as well as AICD peptides, resulting in the proposed feedback cycle. This cycle is closely linked to a feedback mechanism proposed for Aβ generation and degradation. AICD decreases APP processing by downregulating the expression of WASP‐family verprolin homologous protein 1 (*WASF1*), resulting in impaired budding of APP containing vesicles from the Golgi‐apparatus, thereby reducing cell‐surface APP and Aβ generation (Ceglia et al., 2015).

For the understanding of the disease mechanism, it should be taken into consideration that APP processing and therefore Aβ production is a continuous ongoing process under physiological conditions. Obviously, to achieve a homeostasis where no accumulation of Aβ takes place, Aβ‐degradation and production has to be tightly regulated. Our paper might help to understand that this regulation encompasses AICD as a pivotal element both in regulating Aβ‐degradation and Aβ production and importantly also in regulating its own degradation. Under pathological conditions, the disturbance of these complex entangled cycles leads to an accumulation of Aβ and promotes the progression of the disease.

## 4 EXPERIMENTAL PROCEDURES

### 4.1 Chemicals and reagents

All chemicals and reagents were obtained from Merck former Sigma‐Aldrich if not stated otherwise.

### 4.2 Cell culture, mouse and human brain samples

Different MEF and human neuroblastoma cells (SH‐SY5Y) were used for cell‐based experiments. MEF WT, MEF lacking both PS1 and PS2 (MEF PS1/2−/−), APP/ALPL2 deficient MEF (MEF APP/APLP2−/−) and MEF expressing a truncated APP construct lacking the last 15 C‐terminal aa (MEF APPΔCT15) were cultivated in Dulcecco’s Modified Eagle’s Medium (DMEM) containing 10% fetal calf serum (FCS; PAN‐Biotech). For MEF PS1/2−/− cells retransfected with PS1 (MEF PS1res) (Grimm et al., 2005) the culture medium additionally contained 300 μg/ml Zeocin (Fisher Scientific). SH‐SY5Y WT, SH‐SY5Y lacking PS1 (SH‐SY5Y PS1−/−) or APP (SH‐SY5Y APP−/−) due to clustered regularly interspaced short palindromic repeats (CRISPR)/CRISPR associated (Cas) mediated knockout (see below) were maintained in DMEM/10% FCS supplemented with 0.1 mM non‐essential amino acid solution (MEM). For SH‐SY5Y cells overexpressing human APP^695^, hygromycin B (400 μg/ml; PAN‐Biotech) was added to the medium. Zeocin (300 μg/ml; Fisher Scientific) containing DMEM/10% FCS was used for SH‐SY5Y cells stably overexpressing the C‐terminal 50 aa of APP (SH‐SY5Y C50). Validations of the used cell lines are provided in Figure S2.

Samples of murine WT, APP−/− and APPΔCT15 brain tissue were provided by Prof. U. Müller (Institute of Pharmacy and Molecular Biotechnology, University of Heidelberg, Germany).

For the ex vivo gene expression analysis we used two cohorts of human AD *postmortem* brain samples dissected from the prefrontal cortex and provided by The Netherland Brain Bank (Netherlands Institute for Neuroscience, Amsterdam, The Netherlands; NBB). The first cohort includes 121 female and 35 male brain samples with an average *postmortem* delay of 06:06 hours and Braak stages 4–6. The second one includes 36 female and 31 male brain samples with Braak stages 1–3 and an average *postmortem* delay of 06:10 hours (see Table S1).

### 4.3 Generation of SH‐SY5Y APP−/− and PS1−/− cells by CRISPR/Cas9

*CRISPRdirect* was used to design the CRISPR/Cas guide sequences to mediate APP and PS1 KO. Cloning into the pSpCas9(BB)‐2A‐Puro (PX459) plasmid was performed according to Ran and colleagues (Ran et al., 2013). A detailed description can be found in supporting information.

### 4.4 Treatment of cells with inhibitors and AICD peptides

Incubation of cells with γ‐secretase inhibitor DAPT (2.5 μM) and γ‐secretase inhibitor X (2 µM) or the corresponding solvent control DMSO was carried out for 48 h (24 h + 24 h) in DMEM culture medium containing 1% FCS. PPARγ‐inhibitor GW9662 (10 µM) and PGC‐1α‐inhibitor SR‐18292 (20 µM) or DMSO as solvent control were incubated for 16 h (4 h + 12 h) in DMEM containing 1% FCS. For 48 h incubation of cells with 2.5 μM synthetic AICD peptide (KMQQNGYENPTYKFFEQMQN; Genscript) or the solvent H_2_O we used Saint‐PhD protein transfection reagent (Synvolux Therapeutics) according to manufacturer's protocol. Long‐term AICD incubation (>9 days) was performed by changing the medium containing 2 μM AICD every 12 h. Uptake and translocation of AICD to the nucleus was controlled as described in Robinson et al. (2014) (same results of translocation of AICD to the nucleus were observed; data not shown).

### 4.5 Lactate dehydrogenase (LDH) activity assay

Cytotoxicity Detection Kit (LDH) from Roche was used according to the manufacturer’s protocol for measurement of cytotoxicity of the different treatments. No cytotoxicity >5% was detected for any treatment condition.

### 4.6 Transfection of cells with plasmid DNA

Lipofectamine^®^ 2000 Transfection Reagent (Fisher Scientific) and Opti‐MEM (Invitrogen) were used according to manufacturer's protocol for transfections.

For overexpression of the different APP isoforms the following vectors were used: pcDNA™3.1/Zeo^(+)^ APP^695^, pcDNA™3.1/Zeo^(+)^ APP^751^, and pcDNA™3.1/Zeo^(+)^ APP^770^. They were applied to confluent cells on 6‐well plates and further analysis was performed 48 h afterward.

For promoter activity assays confluent cells on 24‐well plates were transfected with the dual reporter system vector pEZX‐PG04‐IDE‐GLuc (GeneCopoeia) 24 h prior to further analysis.

MEF cells were transfected with SureSilencing™‐Insulin‐degrading enzyme shRNA plasmids (SABioscience) according to the manufacturer for IDE‐KD analysis. Further experiments were performed 24 h after transfection.

### 4.7 Protein concentration

Bicinchoninic acid assay was used for determination of the protein concentrations in samples according to Smith et al. (1985) as described in detail earlier. Prior to their use in experiments, samples were adjusted to equal protein amounts.

### 4.8 Total Aβ‐degradation

Degradation of total Aβ in different MEF cell lines was performed according to Grimm et al. (2016) as described in detail in supporting information.

### 4.9 Western blot experiments

For examination of IDE protein level, cell lysates were prepared as described above. Lysis buffer was additionally supplemented with Complete protease inhibitor cocktail (Roche Diagnostics). After centrifugation of the lysates for 5 min at 13,000 *g* and 4°C the supernatants were adjusted to equal protein amounts and loaded on 10–20% tris‐tricine‐gradient gels (Anamed Elektrophorese) and proteins were transferred onto nitrocellulose membranes afterward (Whatman). A detailed description of Western blot analysis including the used antibodies can be found in supporting information. Signal detection was performed with the enhanced chemiluminescence (ECL‐) method (Perkin Elmer) and for densitometrical quantification of band intensity after subtraction of the background signal; Image Gauge version 3.45 software (Fujifilm) was used.

### 4.10 IDE activity assay

The enzyme activity of IDE was measured as published by Miners et al. (2008) with minor modifications as described earlier (Grimm et al., 2016). A detailed overview is given in supporting information.

### 4.11 IDE promoter activity assay

Activity of the IDE promoter was measured by transiently transfecting cells with the dual reporter system vector pEZX‐PG04‐IDE‐GLuc as described before (Grimm et al., 2016). For a detailed description see supporting information.

### 4.12 RT‐PCR experiments

For gene expression analysis quantitative real‐time (RT) polymerase chain reaction (PCR) was performed and results were normalized to β‐*actin* and changes in expression were calculated using the 2^−(ΔΔCt)^ method (Livak & Schmittgen, 2001). A detailed description can be found in supporting information.

### 4.13 Data analysis

The quantified data represent an average of at least five independent experiments for each cell culture experiment. 223 human brain samples were analyzed. For APP−/− mice four brain samples and for APPΔCT15 eight brain samples derived from different mice were analyzed. Error bars represent the standard error of the mean. Prior to calculating statistical significance, it was checked if data are normally distributed via Shapiro–Wilk‐test and Levene's test whether homogeneity of variances could be assumed. If data were normally distributed and variances were homogeneous, statistical significance was calculated via analysis of variances test (ANOVA). If the assumption for homogeneity of variances was violated, statistical significance was calculated via Welch's test. If data were not normally distributed, we used the non‐parametric Kruskal–Wallis *H* test. When more than two groups were compared, pairwise comparison followed via Dunn's post hoc test after significant differences in the Kruskal–Wallis *H* test was obtained. After a significant difference in ANOVA, we either used two‐sided Dunnett post hoc test, or Tukey‐HSD, to calculate statistical differences between groups, for Welch's test we used the Games–Howell post hoc test. For the statistical analysis of the human brain samples, we assumed that the data were normally distributed, since the sample size was over 200 (Ghasemi & Zahediasl, 2012). Correlation coefficients were thus calculated via the Pearson method. Significance was set at *p ≤ 0.05, **p ≤ 0.01 and ***p ≤ 0.001. All calculations were done with IBM SPSS Statistics version 25. Detailed overview of used statistical test can be found in Table S3.

## CONFLICT OF INTEREST

The authors declare no conflicts of interest.

## AUTHOR CONTRIBUTIONS

Lauer, A., Mett, J., Janitschke, D., Thiel, A., Stahlmann, C., Bachmann, C., and Ritzmann F. performed the experiments; Müller. U.C., Riemenschneider M. and Hartmann, T. provided material; Mett, J., Lauer, A., Thiel, A., Stahlmann, C., Schrul, B., Grimm, H.S., and Grimm, M.O.W. wrote the manuscript; Hartmann, T., Stein, R. and Grimm, M.O.W. designed the study.

## ETHICAL APPROVAL

Treatment of WT, APP−/− and APPΔCT15+/− mice followed the German law for the use of laboratory animals (animal welfare act, TierSchG) and the Directive 2010/63/EU. The German administration approved animal housing, breeding and sacrifice. Human *postmortem* brain samples were collected from donors for or from whom a written informed consent for a brain autopsy and the use of the material and clinical information for research purposes had been obtained.

## Data Availability

The data that support the findings of this study are available from the corresponding author upon reasonable request.
