# Supplementary material for: Regulatory feedback cycle of the insulin‐degrading enzyme and the amyloid precursor protein intracellular domain: Implications for Alzheimer’s disease
Source: Aging Cell. 2020 Oct 31;19(11):e13264. doi: 10.1111/acel.13264 (PMC7681056; doi:10.1111/acel.13264)
Supplement: Supplementary file 1 [file ACEL-19-e13264-s001.docx]

**Lauer *et al.* (2020) Supporting Information**

**Supplemental table S1: Overview of NBB brain sample cohort 1 (Braak stages 4-6).** Characteristics including NBB number, sex, age in years, Braak staging, *post mortem* (PM) delay in hours, APOE genotype, amyloid, brain region and protein content in mg per g wet weight.

| **NBB #** | **Sex** | **Age [y]** | **B&B** | **PM**  **delay [h]** | **APOE ε** | **amyloid** | **brain region** | **protein content**  **[mg/g wet weight]** |
| --- | --- | --- | --- | --- | --- | --- | --- | --- |
| **1990_069** | F | 91 | 4 | 04:10 | 43 |  | medial frontal gyrus | 58.92 |
| **1991_078** | F | 98 | 5 | 04:00 | 43 |  | medial frontal gyrus | 79.60 |
| **1991_080** | F | 86 | 4 | 03:30 | 43 |  | medial frontal gyrus | 61.12 |
| **1992_052** | F | 90 | 5 | 05:00 | 43 | A | medial frontal gyrus | 50.20 |
| **1992_068** | F | 78 | 6 | 05:30 | 44 | A | inferior frontal gyrus | 47.64 |
| **1993_138** | F | 74 | 6 | 04:30 | 43 |  | medial frontal gyrus | 59.51 |
| **1994_002** | F | 80 | 6 | 06:40 | 33 |  | inferior frontal gyrus | 79.08 |
| **1994_006** | F | 90 | 5 | 02:35 | 44 |  | inferior frontal gyrus | 75.19 |
| **1994_007** | F | 78 | 6 | 05:00 | 43 |  | inferior frontal gyrus | 84.00 |
| **1994_014** | F | 77 | 5 | 03:35 | 33 |  | inferior frontal gyrus | 83.55 |
| **1994_016** | F | 86 | 5 | 05:15 | 33 |  | inferior frontal gyrus | 53.88 |
| **1994_022** | F | 87 | 5 | 05:30 | 42 |  | inferior frontal gyrus | 80.49 |
| **1994_023** | F | 87 | 6 | 04:00 | 43 |  | inferior frontal gyrus | 59.29 |
| **1994_045** | F | 82 | 6 | 04:00 | 44 |  | inferior frontal gyrus | 69.41 |
| **1994_046** | F | 90 | 4 | 06:15 | 43 |  | inferior frontal gyrus | 70.82 |
| **1994_101** | F | 87 | 6 | 05:00 | 43 |  | medial frontal gyrus | 81.32 |
| **1996_063** | F | 82 | 5 | 09:00 | 43 | C | inferior frontal gyrus | 76.76 |
| **1997_087** | F | 78 | 5 | 03:10 | 43 | C | medial frontal gyrus | 91.12 |
| **1997_133** | F | 95 | 5 | 04:45 | 33 |  | superior frontal gyrus | 52.41 |
| **1997_136** | F | 62 | 6 | 04:25 | 44 |  | superior frontal gyrus | 73.66 |
| **1997_167** | F | 82 | 5 | 04:00 | 44 |  | medial frontal gyrus | 73.48 |
| **1998_007** | F | 75 | 6 | 03:50 | 44 |  | medial frontal gyrus | 79.10 |
| **1998_015** | F | 87 | 6 | 06:15 | 43 |  | medial frontal gyrus | 72.70 |
| **1998_026** | F | 67 | 5 | 03:30 | 44 | C | superior frontal gyrus | 72.85 |
| **1998_032** | F | 92 | 4 | 03:50 | 42 | C | superior frontal gyrus | 63.72 |
| **1998_052** | F | 90 | 4 | 04:00 | 43 | 0 | medial frontal gyrus | 59.78 |
| **1998_065** | F | 90 | 5 | 03:40 | 43 | 0 | medial frontal gyrus | 53.99 |
| **2000_062** | F | 91 | 4 | 03:45 | 43 | A | medial frontal gyrus | 64.29 |
| **2000_119** | F | 85 | 5 | 06:10 | 43 | B | medial frontal gyrus | 85.72 |
| **2000_138** | F | 84 | 5 | 05:15 | 33 | B | medial frontal gyrus | 64.39 |
| **2001_010** | F | 84 | 4 | 04:15 | 32 | B | superior frontal gyrus | 75.03 |
| **2001_013** | F | 68 | 5 | 04:51 | 33 | B | medial frontal gyrus | 91.68 |
| **2001_019** | F | 92 | 5 | 04:55 | 43 | A | superior frontal gyrus | 50.78 |
| **2001_066** | F | 96 | 5 | 05:50 | 33 | C | medial frontal gyrus | 117.68 |
| **2001_070** | F | 86 | 6 | 05:40 | 44 | C | medial frontal gyrus | 91.01 |
| **2001_071** | F | 91 | 5 | 07:50 | 43 | C | medial frontal gyrus | 77.71 |
| **2001_076** | F | 78 | 6 | 03:45 | 44 | C | medial frontal gyrus | 97.31 |
| **2001_081** | F | 93 | 5 | 05:05 | 33 | 0 | medial frontal gyrus | 87.85 |
| **2001_087** | F | 89 | 5 | 03:15 | 43 | 0 | medial frontal gyrus | 69.95 |
| **2001_095** | F | 81 | 6 | 05:30 | 44 | B | medial frontal gyrus | 81.37 |
| **2001_098** | F | 94 | 6 | 05:40 | 43 | B | medial frontal gyrus | 65.86 |
| **2001_105** | F | 79 | 4 | 05:20 | 43 | B | medial frontal gyrus | 87.13 |
| **2001_116** | F | 94 | 4 | 04:20 | 32 | C | medial frontal gyrus | 83.81 |
| **2001_120** | F | 87 | 5 | 04:00 | 43 | C | medial frontal gyrus | 78.89 |
| **2001_124** | F | 86 | 6 | 05:20 | 44 | C | medial frontal gyrus | 71.31 |
| **2001_125** | F | 77 | 6 | 08:30 | 44 | C | medial frontal gyrus | 79.19 |
| **2001_129** | F | 91 | 6 | 03:40 | 33 | C | medial frontal gyrus | 68.28 |
| **2002_001** | F | 84 | 5 | 06:30 | 43 | C | medial frontal gyrus | 85.77 |
| **2002_004** | F | 91 | 4 | 04:15 | 43 | B | medial frontal gyrus | 85.79 |
| **2002_027** | F | 85 | 5 | 05:00 | 44 | C | medial frontal gyrus | 71.70 |
| **2002_047** | F | 83 | 5 | 07:17 | 43 | C | medial frontal gyrus | 81.67 |
| **2002_050** | F | 95 | 4 | 04:10 | 33 | B | medial frontal gyrus | 96.71 |
| **2002_056** | F | 85 | 5 | 03:45 | 43 | C | medial frontal gyrus | 74.04 |
| **2002_061** | F | 76 | 5 | 10:45 | 44 | C | medial frontal gyrus | 65.51 |
| **2002_072** | F | 62 | 6 | 04:45 | 43 | C | medial frontal gyrus | 78.69 |
| **2002_080** | F | 86 | 5 | 04:10 | 44 | C | medial frontal gyrus | 75.86 |
| **2002_085** | F | 79 | 5 | 04:15 | 33 | C | medial frontal gyrus | 98.95 |
| **2002_088** | F | 78 | 5 | 04:00 | 43 | C | medial frontal gyrus | 89.75 |
| **2002_093** | F | 87 | 5 | 09:15 | 33 | C | medial frontal gyrus | 82.55 |
| **2002_096** | F | 82 | 6 | 06:00 | 42 | C | medial frontal gyrus | 51.30 |
| **2002_102** | F | 88 | 5 | 03:15 | 33 | C | medial frontal gyrus | 79.55 |
| **2003_008** | F | 95 | 4 | 03:40 | 43 | C | medial frontal gyrus | 72.72 |
| **2003_034** | F | 91 | 5 | 06:30 | 43 | C | medial frontal gyrus | 89.64 |
| **2003_071** | F | 84 | 5 | 07:15 | 43 | C | medial frontal gyrus | 51.74 |
| **2003_110** | F | 82 | 5 | 04:35 | 43 | C | medial frontal gyrus | 66.30 |
| **2004_006** | F | 89 | 5 | 07:00 | 43 | C | medial frontal gyrus | 99.58 |
| **2004_010** | F | 85 | 5 | 07:00 | 33 | C | medial frontal gyrus | 66.84 |
| **2004_011** | F | 84 | 5 | 05:55 | 33 | C | medial frontal gyrus | 65.55 |
| **2004_025** | F | 90 | 4 | 05:30 | 43 | C | medial frontal gyrus | 52.12 |
| **2004_029** | F | 78 | 5 | 04:50 | 44 | C | medial frontal gyrus | 89.96 |
| **2004_030** | F | 89 | 5 | 04:40 | 43 | C | medial frontal gyrus | 65.50 |
| **2004_034** | F | 89 | 5 | 04:40 | 43 | C | medial frontal gyrus | 58.84 |
| **2004_038** | F | 62 | 5 | 05:55 | 33 | C | medial frontal gyrus | 59.51 |
| **2004_039** | F | 69 | 6 | 04:45 | 33 | C | medial frontal gyrus | 70.15 |
| **2004_043** | F | 88 | 5 | 05:10 | 43 | C | medial frontal gyrus | 59.77 |
| **2004_053** | F | 94 | 4 | 05:05 | 43 | C | medial frontal gyrus | 82.52 |
| **2004_058** | F | 86 | 4 | 05:05 | 43 | C | medial frontal gyrus | 80.20 |
| **2004_064** | F | 88 | 5 | 06:25 | 33 | C | medial frontal gyrus | 75.63 |
| **2004_077** | F | 74 | 5 | 08:25 | 43 | C | medial frontal gyrus | 87.11 |
| **2004_083** | F | 90 | 5 | 04:30 | 42 | B | medial frontal gyrus | 72.32 |
| **2004_086** | F | 84 | 5 | 04:30 | 44 | C | medial frontal gyrus | 66.49 |
| **2005_003** | F | 84 | 5 | 06:20 | 33 | C | medial frontal gyrus | 62.77 |
| **2005_005** | F | 94 | 5 | 04:30 | 33 | C | medial frontal gyrus | 114.23 |
| **2005_011** | F | 93 | 4 | 02:30 | 32 | C | medial frontal gyrus | 75.79 |
| **2005_012** | F | 91 | 6 | 05:45 | 43 | C | medial frontal gyrus | 65.18 |
| **2005_013** | F | 89 | 6 | 04:30 | 43 | C | medial frontal gyrus | 90.85 |
| **2005_016** | F | 81 | 5 | 06:15 | 43 | C | medial frontal gyrus | 79.74 |
| **2005_021** | F | 89 | 5 | 10:20 | 33 | C | medial frontal gyrus | 100.92 |
| **2005_022** | F | 78 | 5 | 04:35 | 43 | C | medial frontal gyrus | 61.14 |
| **2005_023** | F | 81 | 6 | 06:00 | 33 | C | medial frontal gyrus | 75.86 |
| **2005_026** | F | 84 | 5 | 04:50 | 43 | C | medial frontal gyrus | 68.82 |
| **2005_036** | F | 94 | 4 | 06:04 | 43 | C | medial frontal gyrus | 95.73 |
| **2005_064** | F | 77 | 5 | 03:45 | 43 | C | medial frontal gyrus | 65.96 |
| **2005_070** | F | 77 | 5 | 07:00 | 33 | C | medial frontal gyrus | 85.50 |
| **2005_074** | F | 72 | 5 | 09:00 | 43 | C | medial frontal gyrus | 100.77 |
| **2006_006** | F | 87 | 6 | 05:00 | 43 | C | medial frontal gyrus | 75.80 |
| **2006_010** | F | 93 | 4 | 06:45 | 33 | C | inferior frontal gyrus | 51.71 |
| **2006_020** | F | 77 | 5 | 03:05 | 43 | C | medial frontal gyrus | 65.61 |
| **2006_044** | F | 86 | 4 | 05:55 | 43 | B | medial frontal gyrus | 63.96 |
| **2007_089** | F | 74 | 5 | 05:30 | 43 | C | inferior frontal gyrus | 89.46 |
| **2008_004** | F | 82 | 6 | 04:20 | 43 | C | inferior frontal gyrus | 74.89 |
| **2008_018** | F | 84 | 6 | 04:05 | n/a | C | inferior frontal gyrus | 57.15 |
| **2008_045** | F | 85 | 4 | 06:00 | n/a | C | medial frontal gyrus | 51.55 |
| **2008_063** | F | 78 | 5 | 08:25 | n/a | C | medial frontal gyrus | 61.83 |
| **2009_006** | F | 81 | 5 | 06:10 | n/a | C | medial frontal gyrus | 69.60 |
| **2009_009** | F | 88 | 5 | 06:45 | n/a | C | medial frontal gyrus | 63.58 |
| **2009_019** | F | 84 | 4 | 06:00 | n/a | C | medial frontal gyrus | 56.56 |
| **2009_041** | F | 85 | 6 | 05:10 | n/a | C | medial frontal gyrus | 63.26 |
| **2009_049** | F | 81 | 4 | 06:15 | n/a | C | medial frontal gyrus | 68.90 |
| **2009_050** | F | 88 | 5 | 08:15 | n/a | C | medial frontal gyrus | 52.51 |
| **2009_065** | F | 75 | 5 | 05:00 | n/a | C | medial frontal gyrus | 71.19 |
| **2009_069** | F | 72 | 6 | 05:55 | n/a | C | medial frontal gyrus | 91.38 |
| **2009_082** | F | 66 | 5 | 06:30 | n/a | C | medial frontal gyrus | 66.17 |
| **2009_086** | F | 84 | 6 | 04:50 | n/a | C | medial frontal gyrus | 50.44 |
| **2009_088** | F | 90 | 6 | 05:40 | n/a | C | medial frontal gyrus | 68.98 |
| **2009_100** | F | 94 | 4 | 08:05 | n/a | C | medial frontal gyrus | 62.54 |
| **2009_101** | F | 96 | 4 | 04:30 | n/a | C | medial frontal gyrus | 60.66 |
| **2009_105** | F | 82 | 5 | 05:25 | n/a | C | medial frontal gyrus | 66.58 |
| **2010_001** | F | 92 | 5 | 03:25 | n/a | C | medial frontal gyrus | 75.60 |
| **2010_054** | F | 82 | 6 | 05:30 | n/a | C | medial frontal gyrus | 67.60 |
| **2010_069** | F | 66 | 5 | 08:15 | n/a | C | medial frontal gyrus | 47.23 |
| **1990_117** | M | 86 | 5 | 04:10 | 33 |  | medial frontal gyrus | 61.59 |
| **1991_088** | M | 90 | 5 | 04:55 | 43 |  | medial frontal gyrus | 52.64 |
| **1992_084** | M | 76 | 5 | 03:30 | 43 | A | medial frontal gyrus | 64.31 |
| **1992_088** | M | 83 | 4 | 03:40 | 33 | A | medial frontal gyrus | 56.41 |
| **1994_082** | M | 64 | 6 | 05:55 | 44 |  | inferior frontal gyrus | 91.40 |
| **1994_086** | M | 75 | 5 | 05:30 | 43 |  | inferior frontal gyrus | 93.30 |
| **1995_077** | M | 72 | 5 | 04:45 | 33 |  | inferior frontal gyrus | 64.34 |
| **1998_011** | M | 62 | 5 | 03:30 | 43 |  | medial frontal gyrus | 85.87 |
| **1998_132** | M | 75 | 5 | 05:15 | 43 |  | medial frontal gyrus | 64.06 |
| **2001_063** | M | 85 | 5 | 04:45 | 43 | C | superior frontal gyrus | 53.82 |
| **2002_002** | M | 92 | 4 | 03:30 | 33 | C | medial frontal gyrus | 50.07 |
| **2003_070** | M | 87 | 5 | 06:10 | 33 | C | medial frontal gyrus | 64.52 |
| **2004_032** | M | 64 | 5 | 04:45 | 42 | C | medial frontal gyrus | 104.25 |
| **2005_010** | M | 93 | 5 | 04:30 | 43 | C | medial frontal gyrus | 60.84 |
| **2005_028** | M | 93 | 4 | 05:50 | 33 | C | medial frontal gyrus | 59.78 |
| **2005_033** | M | 70 | 5 | 05:35 | 43 | C | medial frontal gyrus | 104.91 |
| **2005_071** | M | 64 | 6 | 07:30 | 33 | C | medial frontal gyrus | 80.05 |
| **2005_075** | M | 82 | 5 | 05:05 | 32 | C | medial frontal gyrus | 66.30 |
| **2006_013** | M | 81 | 4 | 04:50 | 44 | C | medial frontal gyrus | 69.98 |
| **2006_018** | M | 73 | 6 | 06:15 | 43 | C | medial frontal gyrus | 46.39 |
| **2006_048** | M | 70 | 6 | 04:50 | 44 | C | medial frontal gyrus | 61.58 |
| **2006_051** | M | 61 | 5 | 04:00 | 33 | C | medial frontal gyrus | 67.15 |
| **2008_005** | M | 90 | 4 | 04:20 | 43 | B | inferior frontal gyrus | 47.27 |
| **2008_047** | M | 77 | 6 | 06:35 | n/a | C | medial frontal gyrus | 94.07 |
| **2008_075** | M | 88 | 4 | 05:00 | n/a | C | medial frontal gyrus | 80.32 |
| **2009_040** | M | 83 | 6 | 06:10 | n/a | C | medial frontal gyrus | 51.56 |
| **2009_053** | M | 70 | 4 | 04:00 | n/a | C | medial frontal gyrus | 82.23 |
| **2009_059** | M | 74 | 6 | 05:35 | n/a | C | medial frontal gyrus | 74.38 |
| **2009_072** | M | 91 | 4 | 04:10 | n/a | B | medial frontal gyrus | 75.77 |
| **2009_107** | M | 88 | 5 | 04:40 | n/a | C | medial frontal gyrus | 78.31 |
| **2010_004** | M | 77 | 5 | 05:39 | n/a | C | medial frontal gyrus | 57.06 |
| **2010_011** | M | 80 | 4 | 04:00 | n/a | C | medial frontal gyrus | 51.98 |
| **2010_016** | M | 86 | 5 | 06:15 | n/a | C | medial frontal gyrus | 56.41 |
| **2010_051** | M | 74 | 6 | 07:40 | n/a | C | medial frontal gyrus | 57.89 |
| **2010_068** | M | 85 | 4 | 08:35 | n/a | C | medial frontal gyrus | 42.52 |

**Supplemental table S2: Overview of NBB brain sample cohort 2 (Braak stages 1-3).** Characteristics including NBB number, sex, age in years, Braak staging, *post mortem* (PM) delay in hours, APOE genotype, amyloid, brain region and protein content in mg per g wet weight.

| NBB # | Sex | Age [y] | B&B | PM  delay [h] | APOE ε | amyloid | brain region | protein content  [mg/g wet weight] |
| --- | --- | --- | --- | --- | --- | --- | --- | --- |
| 1993_035 | F | 89 | 2 | 04:20 | 33 |  | gyrus rectus | 85.74 |
| 1995_097 | F | 89 | 1 | 06:25 | 43 | B | inferior frontal gyrus | 76.81 |
| 1995_101 | F | 73 | 1 | 05:30 | 33 |  | inferior frontal gyrus | 97.14 |
| 1996_032 | F | 60 | 2 | 08:25 | 43 |  | inferior frontal gyrus | 73.80 |
| 1996_044 | F | 90 | 2 | 05:50 | 33 | A | superior frontal gyrus | 71.21 |
| 1996_051 | F | 71 | 2 | 04:50 | 43 | C | inferior frontal gyrus | 57.18 |
| 1996_078 | F | 87 | 2 | 08:00 | 33 |  | inferior frontal gyrus | 45.61 |
| 1996_084 | F | 78 | 2 | 07:30 | 43 |  | inferior frontal gyrus | 96.02 |
| 1998_016 | F | 82 | 1 | 10:45 | 43 | C | superior frontal gyrus | 71.77 |
| 1998_089 | F | 90 | 1 | 07:15 | 22 |  | superior frontal gyrus | 71.35 |
| 2000_106 | F | 88 | 2 | 05:40 | 33 | B | inferior frontal gyrus | 96.06 |
| 2000_137 | F | 92 | 1 | 07:15 | 32 | B | medial frontal gyrus | 68.30 |
| 2000_142 | F | 82 | 1 | 05:30 | 32 | A | inferior frontal gyrus | 57.14 |
| 2001_006 | F | 91 | 1 | 05:45 | 22 | B | inferior frontal gyrus | 54.54 |
| 2001_029 | F | 90 | 1 | 05:25 | 32 | A | inferior frontal gyrus | 56.02 |
| 2001_079 | F | 90 | 3 | 04:45 | 33 | 0 | inferior frontal gyrus | 83.56 |
| 2001_096 | F | 77 | 1 | 05:40 | 33 | B | inferior frontal gyrus | 82.84 |
| 2001_139 | F | 73 | 2 | 13:35 | 43 | C | inferior frontal gyrus | 70.35 |
| 2002_018 | F | 92 | 1 | 07:00 | 43 | B | inferior frontal gyrus | 85.81 |
| 2002_024 | F | 75 | 1 | 05:30 | 42 | B | inferior frontal gyrus | 54.92 |
| 2003_006 | F | 91 | 3 | 05:20 | 32 | C | inferior frontal gyrus | 85.02 |
| 2004_026 | F | 91 | 1 | 07:45 | 33 | C | inferior frontal gyrus | 87.51 |
| 2004_049 | F | 77 | 1 | 08:20 | 32 | C | inferior frontal gyrus | 88.59 |
| 2005_083 | F | 85 | 1 | 05:00 | 33 | C | inferior frontal gyrus | 58.33 |
| 2006_008 | F | 85 | 2 | 04:40 | 43 | C | inferior frontal gyrus | 79.41 |
| 2006_080 | F | 89 | 2 | 06:25 | 32 | C | medial frontal gyrus | 72.70 |
| 2007_032 | F | 87 | 3 | 07:20 | n/a | C | superior frontal gyrus | 71.64 |
| 2008_105 | F | 89 | 3 | 03:52 | n/a | C | medial frontal gyrus | 69.70 |
| 2009_021 | F | 99 | 2 | 04:15 | n/a | C | medial frontal gyrus | 52.85 |
| 2009_022 | F | 77 | 1 | 02:55 | n/a | C | medial frontal gyrus | 72.72 |
| 2009_095 | F | 71 | 1 | 07:10 | n/a | C | inferior frontal gyrus | 54.66 |
| 2010_007 | F | 85 | 2 | 05:19 | n/a | C | medial frontal gyrus | 74.00 |
| 2010_015 | F | 73 | 1 | 07:45 | n/a | C | medial frontal gyrus | 59.60 |
| 2010_039 | F | 60 | 1 | 06:50 | n/a | B | inferior frontal gyrus | 56.67 |
| 2010_062 | F | 94 | 1 | 05:50 | n/a | C | medial frontal gyrus | 85.85 |
| 2010_070 | F | 60 | 1 | 07:30 | n/a | C | medial frontal gyrus | 56.82 |
| 1990_042 | M | 76 | 1 | 06:00 | 33 |  | orbital gyrus | 36.96 |
| 1991_125 | M | 61 | 1 | 05:40 | 43 |  | gyrus rectus | 70.83 |
| 1992_026 | M | 83 | 1 | 06:25 | 33 |  | gyrus rectus | 114.22 |
| 1992_029 | M | 79 | 2 | 05:10 | 33 | A | lateral orbital gyrus | 37.62 |
| 1993_015 | M | 75 | 1 | 04:15 | 33 | B | orbital gyrus | 107.49 |
| 1994_053 | M | 83 | 1 | 08:50 | 33 |  | orbital gyrus | 60.12 |
| 1994_076 | M | 78 | 2 | 08:25 | 33 |  | orbital gyrus | 69.34 |
| 1995_093 | M | 78 | 1 | 07:00 | 33 |  | inferior frontal gyrus | 75.55 |
| 1996_085 | M | 84 | 1 | 09:00 | 33 |  | inferior frontal gyrus | 80.72 |
| 1996_125 | M | 93 | 3 | 10:25 | 43 |  | superior frontal gyrus | 77.94 |
| 1997_039 | M | 87 | 3 | 04:00 | 33 | C | inferior frontal gyrus | 81.57 |
| 1998_039 | M | 85 | 2 | 04:35 | 33 | B | superior frontal gyrus | 56.10 |
| 1998_049 | M | 87 | 2 | 07:25 | 33 | 0 | superior frontal gyrus | 79.06 |
| 2000_030 | M | 82 | 2 | 13:35 | 33 | A | medial frontal gyrus | 72.84 |
| 2001_017 | M | 79 | 1 | 07:40 | 43 | A | inferior frontal gyrus | 64.11 |
| 2001_021 | M | 82 | 1 | 07:40 | 33 | 0 | inferior frontal gyrus | 56.63 |
| 2001_046 | M | 88 | 1 | 07:25 | 33 | C | inferior frontal gyrus | 82.87 |
| 2001_086 | M | 88 | 1 | 07:00 | 32 | 0 | inferior frontal gyrus | 74.95 |
| 2001_094 | M | 86 | 2 | 05:30 | 43 | B | inferior frontal gyrus | 84.30 |
| 2004_020 | M | 96 | 1 | 05:23 | 33 | C | inferior frontal gyrus | 76.00 |
| 2005_055 | M | 84 | 1 | 07:05 | 33 | C | inferior frontal gyrus | 83.04 |
| 2005_060 | M | 91 | 1 | 08:00 | 33 | C | inferior frontal gyrus | 86.98 |
| 2005_073 | M | 87 | 3 | 06:05 | 33 | C | inferior frontal gyrus | 95.13 |
| 2007_007 | M | 84 | 1 | 05:35 | 33 | C | superior frontal gyrus | 65.72 |
| 2007_046 | M | 89 | 1 | 09:20 | 33 | C | superior frontal gyrus | 53.83 |
| 2008_032 | M | 71 | 2 | 08:55 | n/a | C | medial frontal gyrus | 71.89 |
| 2008_103 | M | 80 | 1 | 08:10 | n/a | C | medial frontal gyrus | 44.96 |
| 2009_001 | M | 88 | 2 | 04:43 | n/a | C | medial frontal gyrus | 81.22 |
| 2009_003 | M | 62 | 1 | 07:20 | n/a | C | medial frontal gyrus | 65.16 |
| 2009_039 | M | 82 | 3 | 12:55 | n/a | C | medial frontal gyrus | 72.52 |
| 2009_075 | M | 88 | 3 | 07:00 | n/a | B | inferior frontal gyrus | 68.98 |

**Supplemental table S3: Overview of the used statistical tests for calculation of significance.**

| Fig. | tested hypothesis | normal  distribution | homogeneity of variance | statistical test |
| --- | --- | --- | --- | --- |
| 1 A | MEF PS1res vs. MEF WT | yes | yes | ANOVA |
| 1 A | MEF PS1res vs. MEF PS1/2-/- | yes | yes | ANOVA |
| 1 B | MEF PS1res + IDE-KD vs. MEF PS1/2-/- + IDE-KD | yes | yes | ANOVA |
| 1 C | MEF WT vs. MEF APP/APLP2-/- | yes | no | Welch-test |
| 1 C | MEF WT vs. MEF APPΔCT15 | yes | no | Welch-test |
| 2 A | MEF PS1res vs. MEF PS1/2-/- | yes | yes | ANOVA |
| 2 B | MEF WT vs. MEF APP/APLP2-/- | no | / | Kruskal-Wallis-test |
| 2 B | MEF WT vs. MEF APPΔCT15 | no | / | Kruskal-Wallis-test |
| 2 C | MEF PS1res vs. MEF PS1/2-/- vs. MEF PS1res + DAPT (multi comparison) | yes | yes | Tukey-HSD |
| 2 D | MEF WT vs. MEF APP/APLP2-/- | yes | yes | ANOVA |
| 2 D | MEF WT vs. MEF APPΔCT15 | yes | no | Welch-test |
| 3 A | MEF PS1res vs. MEF PS1/2-/- | no | / | Kruskal-Wallis-test |
| 3 B | MEF WT vs. MEF APP/APLP2-/- | no | / | Kruskal-Wallis-test |
| 3 B | MEF WT vs. MEF APPΔCT15 | no | / | Kruskal-Wallis-test |
| 3 C | SH-SY5Y WT vs. SH-SY5Y PS1-/- | no | / | Kruskal-Wallis-test |
| 3 D | SH-SY5Y WT vs. SH-SY5Y APP-/- | no | / | Kruskal-Wallis-test |
| 3 D | SH-SY5Y WT vs. SH-SY5Y APP^695^ | no | / | Kruskal-Wallis-test |
| 3 E | MEF APP/APLP2-/- vs. MEF APP/APLP2-/- + APP^695^ vs. MEF APP/APLP2-/- + APP^751^ vs. MEF APP/APLP2-/- + APP^770^ vs. MEF WT (multi comparison) | no | / | Kruskal-Wallis-test / Dunn´s pairwise comparison |
| 4 A | MEFAPPΔCT15 control vs. MEFAPPΔCT15 + C50 vs. MEFAPPΔCT15 + AICD (48h) + MEFAPPΔCT15 + AICD (9d) (multi comparison) | no | / | Kruskal-Wallis-test / Dunn´s pairwise comparison |
| 4 B | SH-SY5Y control vs. SH-SY5Y + C50 | no | / | Kruskal-Wallis-test |
| 4 C | MEFAPPΔCT15 control vs. MEFAPPΔCT15 + C50 | yes | yes | ANOVA |
| 4 C | MEFAPPΔCT15 control vs. MEFAPPΔCT15 + C50 vs. MEFAPPΔCT15 + AICD (48h) | yes | yes | ANOVA |
| 4 D | MEF PS1res vs. MEF PS1/2-/- vs. MEF PS1/2-/- + C50 (multi comparison) | yes | yes | Games-Howell-test |
| 5 A | MEF WT vs. MEF APP/APLP2-/- | no | / | Kruskal-Wallis-test |
| 5 A | MEF WT vs. MEF APPΔCT15 | yes | no | Welch-test |
| 5 B | MEF APPΔCT15 control vs. MEF APPΔCT15 + C50 | yes | yes | ANOVA |
| 6 A | brain WT mice vs. brain APP-/- mice | no | / | Kruskal-Wallis-test |
| 6 A | brain WT mice vs. brain APPΔCT15+/- mice | no | / | Kruskal-Wallis-test |
| 6 B | brain WT mice vs. brain APPΔCT15+/- mice | yes | yes | ANOVA |
| 6 C | Correlation APP gene expression vs. IDE gene expression | no | / | Spearman correlation coefficient |
| 6 D | Correlation APP gene expression vs. IDE gene expression | yes | / | Pearson correlation coefficient |
| S2B | MEF PS1res vs. MEF PS1res + IDE-KD | no | / | Kruskal-Wallis-test |
| S2C | MEF WT vs. MEF APP/APLP2-/- | no | / | Kruskal-Wallis-test |
| S2C | MEF WT vs. MEF APP/APLP2-/- | no | / | Kruskal-Wallis-test |
| S2D | MEF WT vs. MEF APPΔCT15 | no | / | Kruskal-Wallis-test |
| S2E | SH-SY5Y WT vs. SH-SY5Y APP^695^ | no | / | Kruskal-Wallis-test |
| S2F | MEF WT vs. MEF APP/APLP2-/- + APP^695^ vs. MEF APP/APLP2-/- + APP^751^ vs. MEF APP/APLP2-/- + APP^770^ (multi comparison) | no | / | Kruskal-Wallis-test / Dunn´s pairwise comparison |
| S2I | MEF WT vs. MEF APPΔCT15 + C50 | no | / | Kruskal-Wallis-test |
| S2J | SH-SY5Y WT vs. SH-SY5Y + C50 | no | / | Kruskal-Wallis-test |
| S4A | MEF WT vs. MEF APP/APLP2-/- | yes | no | Welch-test |
| S4B | MEF WT vs. MEF APP/APLP2-/- | yes | yes | ANOVA |
| S4C | MEF WT vs. MEF APP/APLP2-/- | yes | yes | ANOVA |
| S4D | MEF WT vs. MEF APP/APLP2-/- | yes | yes | ANOVA |
| S5A | Correlation APP gene expression vs. IDE gene expression | assumed (sample size > 200 (Ghasemi & Zahediasl, 2012) | / | Pearson correlation coefficient |
| S5B | Correlation APP protein level vs. IDE protein level | yes | / | Pearson correlation coefficient |
| S5C | gene expression Braak I vs. Braak II or Braak III or Braak IV or Braak VI (multi comparison) | assumed (sample size > 200 (Ghasemi & Zahediasl, 2012) | yes | ANOVA / Dunnetts test |
| S5C | age Braak I vs. Braak II or Braak III or Braak IV or Braak VI (multi comparison) | assumed (sample size > 200 (Ghasemi & Zahediasl, 2012) | yes | ANOVA (no significant differences) |
| S5D | gene expression Amyloid O vs. Amyloid A or Amyloid B or Amyloid C (multi comparison) | assumed (sample size > 200 (Ghasemi & Zahediasl, 2012) | yes | ANOVA (no significant differences) |
| S5D | age Amyloid O vs. Amyloid A or Amyloid B or Amyloid C (multi comparison) | assumed (sample size > 200 (Ghasemi & Zahediasl, 2012) | yes | ANOVA (no significant differences) |
| S5E | Correlation APP gene expression vs. IDE gene expression female | yes | / | Pearson correlation coefficient |
| S5E | Correlation APP gene expression vs. IDE gene expression male | yes | / | Pearson correlation coefficient |
| S5F | gene expression ApoE22 vs. ApoE32 or ApoE33 or ApoE42 or ApoE43 or ApoE44 (multi comparison) | assumed (sample size > 200 (Ghasemi & Zahediasl, 2012) | yes | ANOVA (no significant differences) |
| S5F | age ApoE22 vs. ApoE32 or ApoE33 or ApoE42 or ApoE43 or ApoE44 (multi comparison) | assumed (sample size > 200 (Ghasemi & Zahediasl, 2012) | yes | ANOVA (no significant differences) |
| S5G | Correlation gene expression vs. age | assumed (sample size > 200 (Ghasemi & Zahediasl, 2012) | / | Pearson correlation coefficient |
| S5H | Correlation gene expression vs. *post mortem* delay | assumed (sample size > 200 (Ghasemi & Zahediasl, 2012) | / | Pearson correlation coefficient |
| S6B | MEF WT vs. MEF APPΔCT15; MEF WT + inhibitor vs. MEF APPΔCT15 + inhibitor; magnitude of effects of both cell lines + Inhibitor vs. – Inhibitor (multi comparison) | no | / | Kruskal-Wallis-test / Dunn´s pairwise comparison |
| S6C | MEF WT vs. MEF APPΔCT15; MEF WT + inhibitor vs. MEF APPΔCT15 + inhibitor; magnitude of effects of both cell lines + Inhibitor vs. – Inhibitor (multi comparison) | no | / | Kruskal-Wallis-test / Dunn´s pairwise comparison |

**Detailed description of the used methods**

Detailed description of generation of SH-SY5Y APP-/- and PS1-/- cells by CRISPR/Cas9

The sequences for mediating APP KO (5’-GCT GGC CGC CTG GAC GGC TC-3’) and PS1 KO (5’-TGG AAG TAG GAC AAC GGT GC-3‘) were cloned into the pSpCas9(BB)-2A-Puro (PX459) plasmid, a gift from Feng Zhang (Addgene plasmid #48139), according to Ran and colleagues (Ran et al., 2013). To generate APP-/- and PS1-/- cells, SH-SY5Y WT cells were transfected with the modified plasmids and with the empty vector to create control cells using Lipofectamine^®^ 2000 Transfection Reagent (see below). Selection of transfected cells was performed with 2μg/ml Puromycin (Fisher Scientific, Schwerte, Germany) and single cell clones were isolated. *APP*- and *PS1*-knock-outs were verified by sequencing the genomic Cas9 guide-RNA binding site and western blot analysis. Sequencing analysis showed a deletion of 16 base pairs near the 3´-terminus of *APP695* exon 1, leading to a translation termination after 35 aa (see supplemental figure S2G). In case of *PS1* the first six codons remained unaffected and subsequently a section of 215 base pairs of the vector integrated into the guideRNA target region. This mutated sequence codes for a protein of 93 aa (see supplemental figure S2H). APP and PS1 were undetectable by western blot analysis in lysates of SH-SY5Y APP-/- and SH-SY5Y PS1-/- cells respectively (see supplemental figure S2G + H).

Detailed description of total Aβ-degradation

Cell lysates were prepared on ice by washing cells with ice-cold PBS and addition of lysis buffer containing 150mM NaCl, 50mM Tris-HCl (pH7.4), 2mM EDTA, 0.1% NP-40 and 0.1% Triton-X 100 for 1h. 60 µg total protein of each sample was incubated with 1 μg/ml human synthetic Aβ_40_ (B. Penke, Szeged, Hungary) in PBS at 37°C for 1h under gentle shaking. For determination of specific IDE- or NEP-mediated Aβ-degradation, 10μM insulin, 10μM thiorphan or a combination of both were added additionally to the samples. Remaining, not degraded human Aβ_40_ was detected by WB analysis using the W02 antibody as described below. Since W02 antibody binds specifically to human Aβ, endogenous murine Aβ peptides of the MEF cells were not detected (see Fig. S3B).

Used antibodies in western blot experiments

**Supplemental table S4: Antibodies used in Western blot analysis.**

| label | epitop | dilution | 2^nd^ antibody |
| --- | --- | --- | --- |
| ST1120 | IDE | 1:2000 | anti-rabbit IgG HRP (W4011; Promega) |
| 67106-1-Ig | IDE | 1:5000 | anti-mouse IgG HRP (P0260; Dako) |
| WO2 | Aβ / APP | 5 µg/ml | anti-mouse IgG HRP (P0260; Dako) |
| sc-7860 H70 | Presenilin 1 | 1:500 | anti-rabbit IgG HRP (W4011; Promega) |
| A5441 | β-actin | 1:5000 | anti-mouse IgG HRP (P0260; Dako) |

Western blot analysis of PS1 and APP for verification of CRISPR/Cas-mediated gene knock-out was conducted as described earlier by our group (Grimm et al., 2015).

Detailed description of IDE activity assay

Anti-IDE antibody ST1120 (5μg/ml) was coated on a Nunc MaxiSorp 96-well plate for 24h and afterwards the plate was washed and then blocked with 10% fatty acid free bovine serum albumin. After an additional washing step, the samples were incubated in the precoated plate for one hour at 20°C and 150rpm, followed by several washing steps. A pre-incubation for 15 min with assay buffer containing 50mM Tris-HCl pH7.4, 1M NaCl, 10μM MgCl_2_, β-secretase inhibitor II, γ-secretase inhibitor IV and complete protease-inhibitor without EDTA was performed prior to addition of the fluorogenic peptide substrate Mca-RPPGFSAFK(Dnp)-OH (10μM). The resulting fluorescence was detected at an excitation wavelength of 320 ± 10nm and an emission wavelength of 405 ± 10nm using a Safire^2^ Fluorometer (Tecan, Crailsheim, Germany).

Detailed description of IDE promoter activity assay

Within the dual reporter system vector pEZX-PG04-IDE-GLuc the *Gaussia* luciferase gene (GLuc) acts as reporter gene since its expression is regulated by the IDE promoter region. Additionally, a tracking gene encoding for secreted alkaline phosphatase (SEAP) is constitutively expressed. The Secrete-Pair Dual Luminescence Assay Kit (GeneCopoeia, Rockville, Maryland, USA) was used according to manufacturer´s protocol to measure the activities of GLuc and SEAP in the conditioned culture medium 48h after transient transfection. The luminescence detection was performed in an Infinite M1000Pro-Fluorometer/Luminometer (Tecan, Crailsheim, Germany). In order to eliminate variations in cell confluency or transfection efficiency, the signal of GLuc activity was normalized to the signal of SEAP activity for each sample.

Detailed description of RT-PCR experiments

Total RNA was extracted from cells and tissues by using the TRIzol Reagent (Fisher Scientific, Schwerte, Germany). Brain samples were stored in liquid nitrogen and for extraction of RNA they were slowly defrosted in TRIzol on ice and afterwards treated by Minilys (Peqlab, Erlangen, Germany) for 30 s. After an incubation of five minutes, RNA was extracted as described in the manufacturer´s protocol. The High-Capacity cDNA Reverse Transcription Kit (Fisher Scientific, Schwerte, Germany) was applied for reverse transcription of 2μg RNA of each sample and the remaining RNA was stored in TRIzol Reagent. In cells transiently transfected with different APP isoforms, isolated RNA was treated with rDNase (Macherey-Nagel, Düren, Germany) according to manufacturer´s instructions to digest DNA. RT-PCR analysis was performed with the Fast SYBR Green Master Mix (Applied Biosystems, Foster City, CA, USA) in a PikoReal Real-Time PCR System (Fisher Scientific, Schwerte, Germany). The used primers were obtained from Eurofins (MWG Operon, Eberberg, Germany) and are listed in table S5.

**Supplemental table S5: Sequences of the used primers.**

| gene | forward-primer 5`-3` | reverse-primer 5`- 3` |
| --- | --- | --- |
| human *β-actin* | CTT CCT GGG CAT GGA GTC | AGC ACT GTG TTG GCG TAC AG |
| murine *β-actin* | CCT AGG CAC CAG GGT GTG AT | TCT CCA TGT CGT CCC AGT TG |
| human *IDE* | TGC CCT AGA CAG GTT TGC AC | CTC CAG GCA TCA TTC ATC ACA T |
| murine *IDE* | GCT ACG TGC AGA AGG ACC TC | TGG ACG TAT AGC CTC GTG GT |
| human *APP* | GGC AGT TAT CCA GCA TTT CC | ATT GAG CAT GGC TTC CAC TC |
| murine *APP* | CCG TTG CCT AGT TGG TGA GT | GTG CCA GTG AAG ATG GGT CT |
| murine *APLP2* | TGT TAA AGC TGT CTG CTC CCA | TAA AGC GTA CGC ACT TTC CC |
| murine & human *C50* | AAA CAG TAC ACA TCC ATC CAT | CTA GTT CTG CAT TTG CTC AAA G |
| human *PGC-1α* | GTT CAA GAT CGC CCT ACA GC | CCC TCT CAG ACT CTC GCT TC |
| murine *PS1* | AAA CAG CCC TGC ACT CGA T | TGG TTG TGT TCC AGT CTC CA |
| murine *PS2* | TCA TGC TAT TTC GTG CCT GTC | GTG TAG ATG AGC TGC CCG TT |

**Supplemental figures**

**
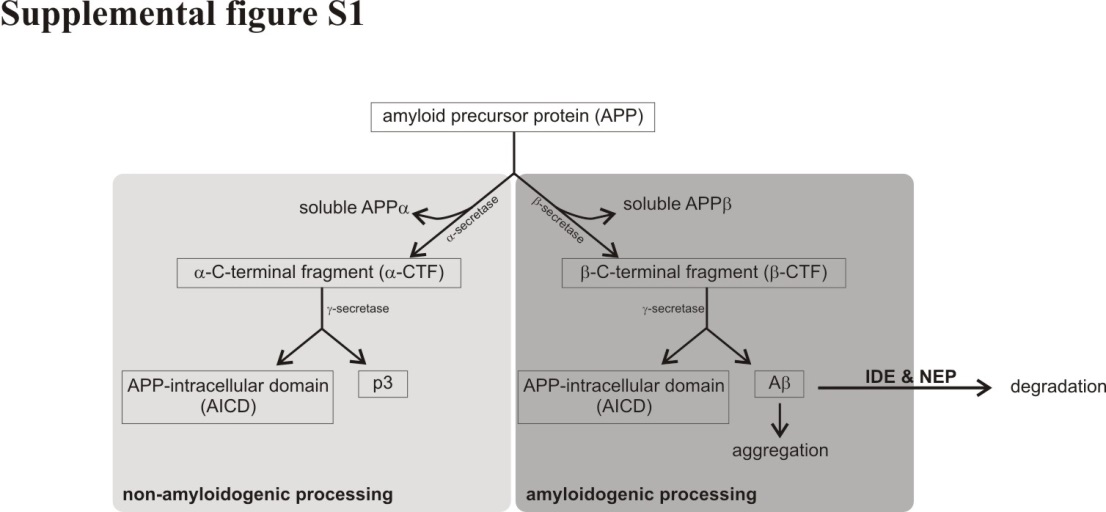
**

**Supplemental figure S1: Schematic overview of APP processing.**

**
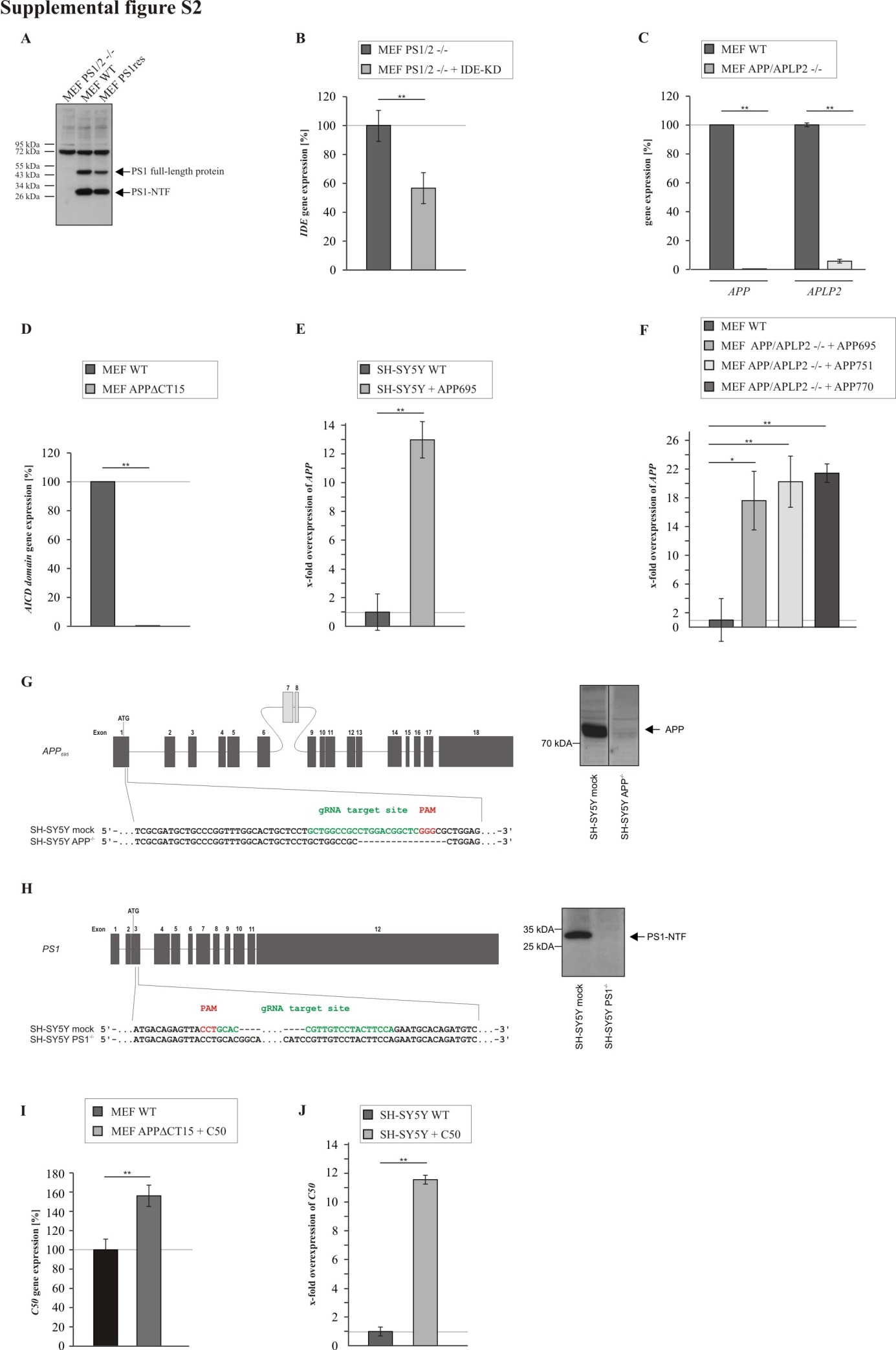
**

**Supplemental figure S2: Validation of the used cell lines. A)** Verification of presenilin 1 retransfection in MEF PS1/2 -/- cells by Western blot analysis. In accordance to literature (Grimm et al., 2005; Herreman et al., 2000). **B)** Reduced *IDE* gene expression in MEF PS1/2 –/- + IDE-KD cells. **C)** Reduced expression of APP and APLP2 in MEF APP/APLP2-/- cells compared to MEF WT cells. In accordance to literature (Heber et al., 2000). **D)** Reduced expression of the AICD domain in MEF APPΔCT15 cells compared to MEF WT cells. In accordance to literature (Ring et al., 2007). **E)** X-fold overexpression of APP^695^ in transfected SH-SY5Y cells compared to SH-SY5Y WT cells. **F)** X-fold overexpression of APP^695^, APP^751^ and APP^770^ in transfected MEF APP/APLP2 -/- cells compared to MEF WT cells. No significant difference was observed between the APP isoforms. **G)** Schematic drawing of APP genomic region. Alternative splicing affecting exons 7 and 8 leads to *APP* mRNA coding for the APP695 isoform. The CRISPR/Cas guide RNA binding site is located at the 3´-end of exon 1 and presented in green adjacent to the protospacer adjacent motif (PAM) sequence in red. The corresponding sequence in SH-SY5Y APP-/- is shown below. Lines indicate deletions of bases. Western blot analysis confirmed the deletion of APP in the knock-out cell line. **H)** The genomic region of PS1 is pictured. The guide RNA target site in exon 3 is magnified and shown in green in vicinity to the PAM sequence in red. The mutated sequence in SH-SY5Y PS1-/- is written below. Lines indicate absent homologous bases. 215 base pairs, which were integrated at the mutation site, are abbreviated by dots. PS1 N-terminal fragment was undetectable by Western blot analysis in lysates of SH-SY5Y PS1-/- cells. **I)** Overexpression of C50 in transfected MEF APPΔCT15 cells compared to MEF WT cells. **J)** X-fold overexpression of C50 in transfected SH-SY5Y cells compared to SH-SY5Y WT cells.


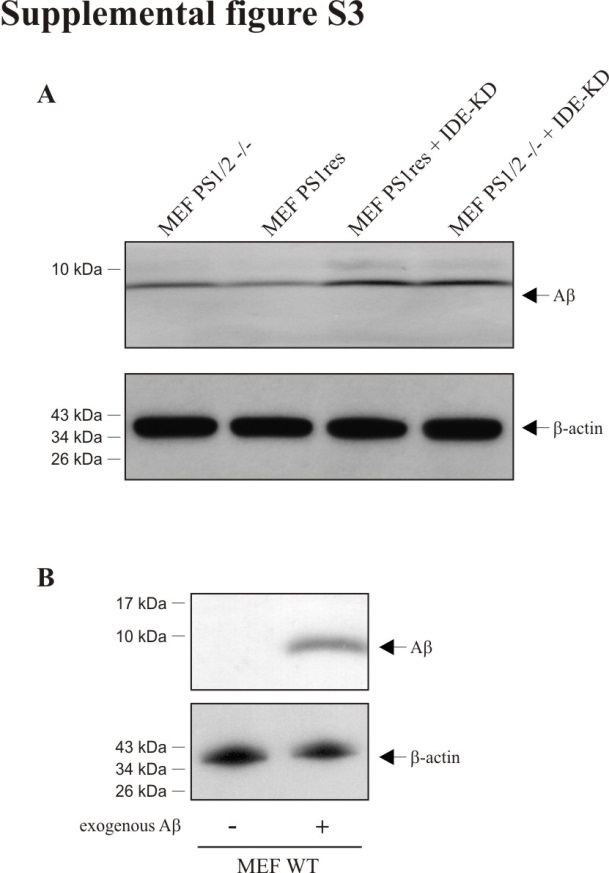


**Supplemental figure S3: Effect of IDE-KD in MEF PS1res and MEF PS1/2 -/- cells on Aβ degradation example blot and validation of Aβ degradation assay in respect to endogenous Aβ detection. A)** Western blot analysis of MEF PS1res + IDE-KD cells and MEF PS1/2 -/- + IDE-KD cells compared to MEF PS1res cells and MEF PS1/2 -/- cells. In presence of IDE-KD, remaining Aβ is elevated; moreover the magnitude of effect between PS1res and PS1/2 -/- is in presence of IDE-KD less pronounced compared to the effect strength without the IDE-KD between the two cell lines. **B)** Validation of W02 detection of exogenous and not endogenous Aβ in mouse embryonic fibroblasts.

**
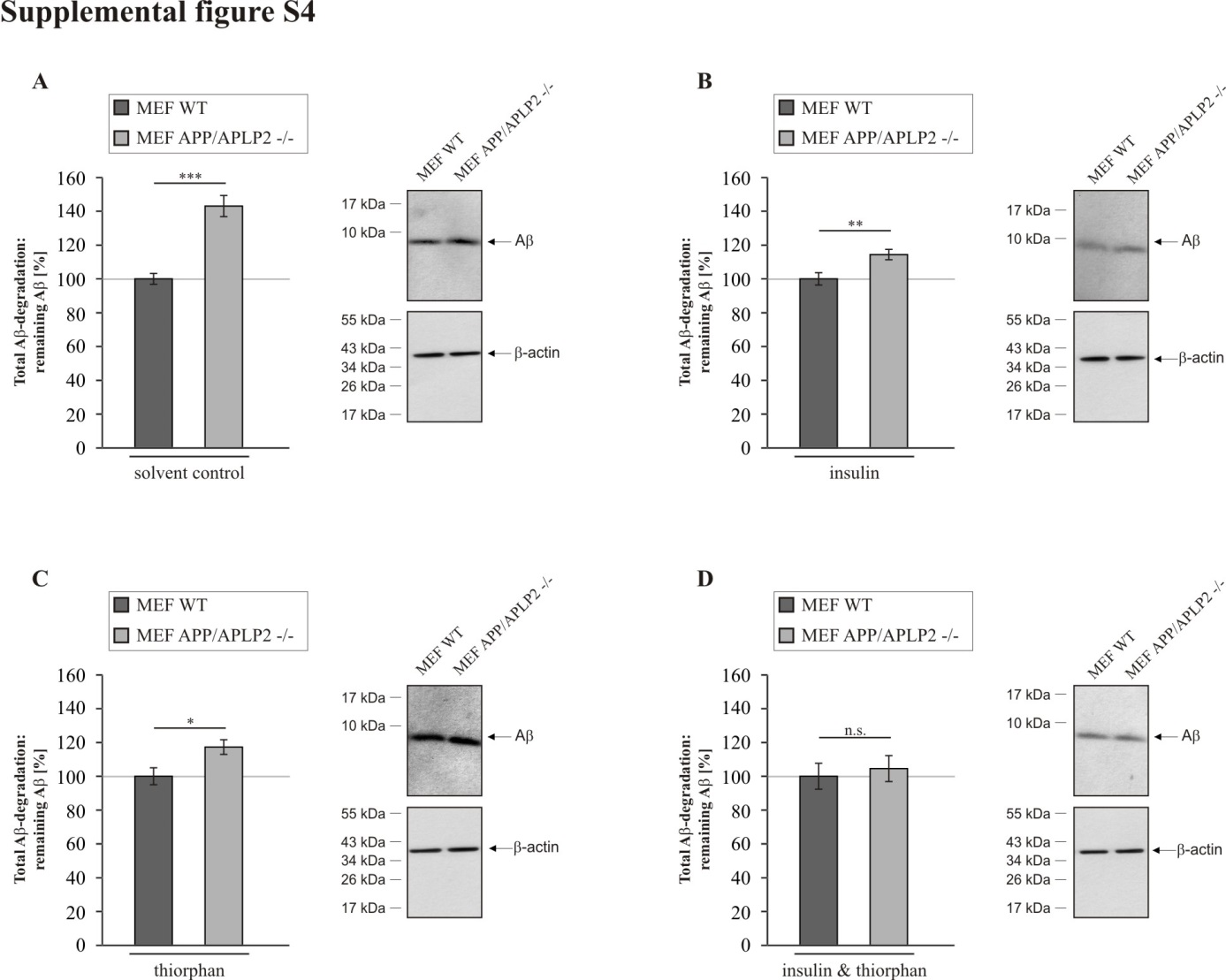
**

**Supplemental figure S4: Aβ-degradation in presence of IDE inhibitor (insulin) and NEP inhibitor (thiorphan) and combination of both inhibitors of MEF WT and MEF APP/APLP2 -/- cells. A)** Solvent control. **B)** Insulin. **C)** Thiorphan. **D)** Insulin and thiorphan. No significant differences in β-actin signals exist between the two compared cell lines (solvent control MEF APP/APLP2 -/-: 90.4%, p=0.357; insulin MEF APP/APLP2 -/-: 99.3%, p=0.970; thiorphan MEF APP/APLP2 -/-: 102.0%, p=0.902; combination of insulin and thiorphan MEF APP/APLP2 -/-: 93.4%, p=0.622). Statistical significance was calculated as described in table S3. Error bars represent the standard error of the mean and significance was set at * p≤0.05, ** p≤0.01 and *** p≤0.001.

**
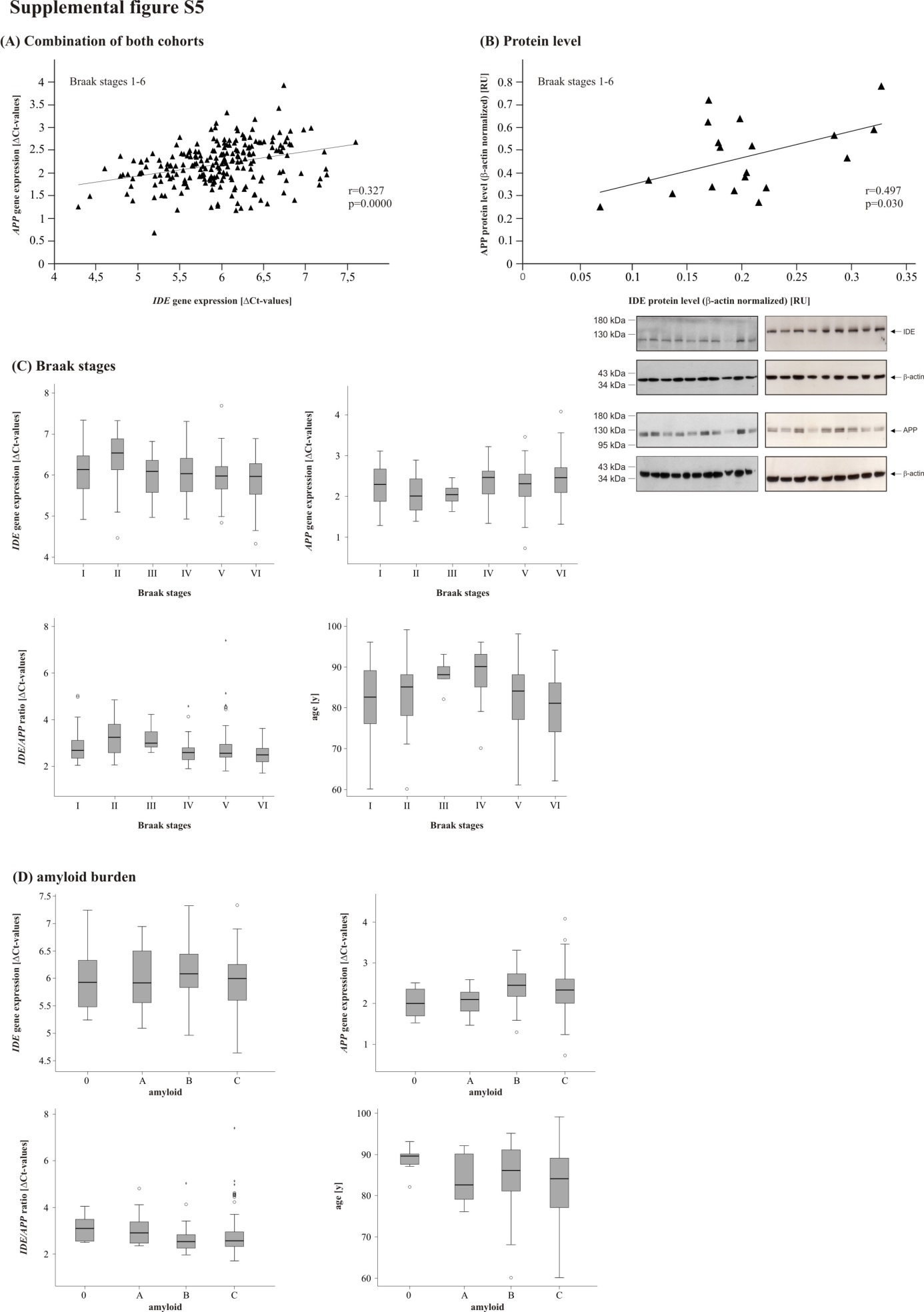
**

**
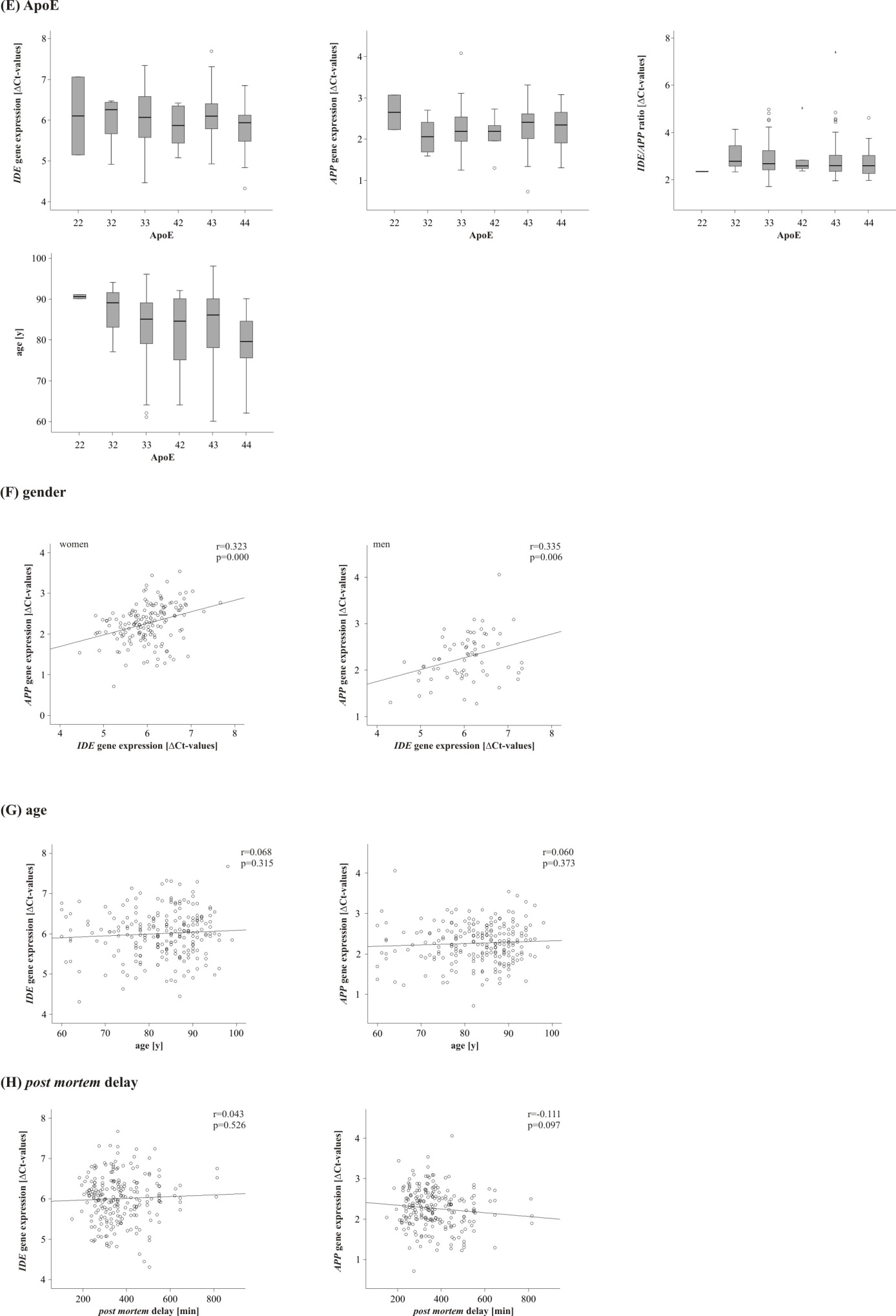
**

**Supplemental figure S5: APP and IDE protein and gene expression of human brain samples. A)** Combination of cohort 1 (Braak 4-6) and cohort 2 (Braak 1-3) of correlation between APP and IDE gene expression. **B)** Correlation of IDE and APP protein level of human brain samples with sufficient protein amount for western blotting. Subgroup analysis of all brain samples in respect of Braak stages **(C)**, amyloid burden **(D)**, ApoE status **(E)**, in respect to IDE, APP and IDE/APP gene expression. Correlation of APP and IDE gene expression in dependence of gender **(F)**. Correlation of IDE gene expression or APP gene expression with age **(G)**. Correlation between IDE and APP gene expression with *post mortem* delay **(H)**.


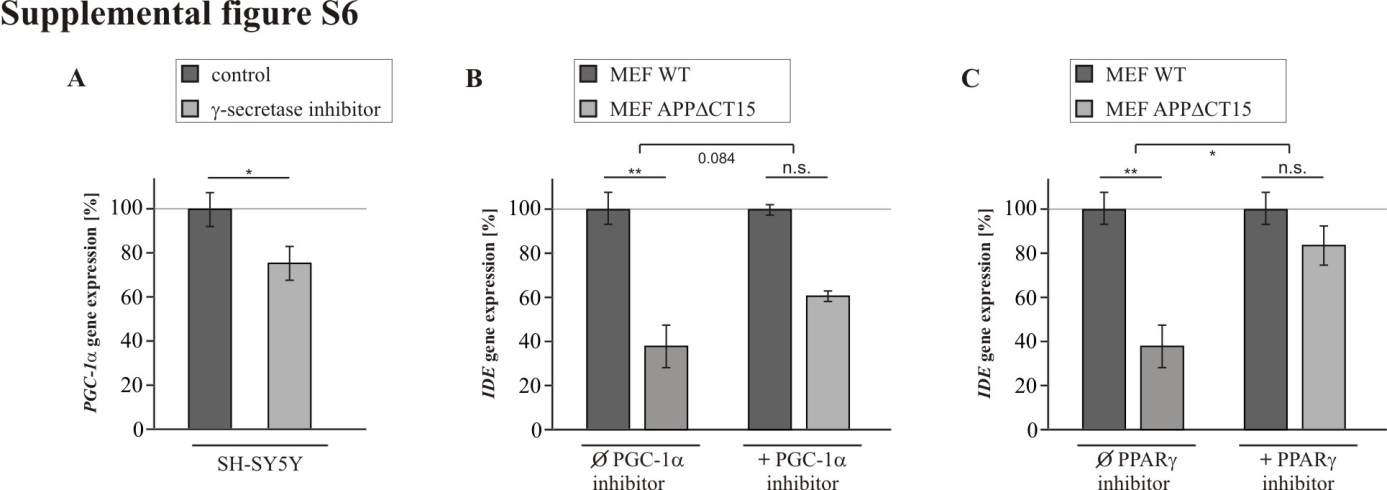


**Supplemental figure S6: APP processing and PGC-1α or PPARγ. A)** *PGC-1a* gene expression in SH-SY5Y cells in presence of y-secretase inhibitor. IDE gene expression in MEF WT and MEF APPΔCT15 cells in presence and absence of PGC-1α inhibitor **(B)** or PPARγ inhibitor **(C).** Statistical significance was calculated as described in table S3. Error bars represent the standard error of the mean and significance was set at * p≤0.05, ** p≤0.01 and *** p≤0.001.

References:

Ghasemi, A., & Zahediasl, S. (2012). Normality tests for statistical analysis: a guide for non-statisticians. *Int J Endocrinol Metab, 10*(2), 486-489. doi:10.5812/ijem.3505

Grimm, M. O., Grimm, H. S., Pätzold, A. J., Zinser, E. G., Halonen, R., Duering, M., . . . Hartmann, T. (2005). Regulation of cholesterol and sphingomyelin metabolism by amyloid-beta and presenilin *Nat Cell Biol, 7*(11), 1118-1123. doi:10.1038/ncb1313

Grimm, M. O., Stahlmann, C. P., Mett, J., Haupenthal, V. J., Zimmer, V. C., Lehmann, J., . . . Hartmann, T. (2015). Vitamin E: Curse or Benefit in Alzheimer's Disease? A Systematic Investigation of the Impact of alpha-, gamma- and delta-Tocopherol on Ass Generation and Degradation in Neuroblastoma Cells. *J Nutr Health Aging, 19*(6), 646-656. doi:10.1007/s12603-015-0506-z

Heber, S., Herms, J., Gajic, V., Hainfellner, J., Aguzzi, A., Rulicke, T., . . . Muller, U. (2000). Mice with combined gene knock-outs reveal essential and partially redundant functions of amyloid precursor protein family members. *J Neurosci, 20*(21), 7951-7963.

Herreman, A., Serneels, L., Annaert, W., Collen, D., Schoonjans, L., & De Strooper, B. (2000). Total inactivation of gamma-secretase activity in presenilin-deficient embryonic stem cells. *Nat Cell Biol, 2*(7), 461-462. doi:10.1038/35017105

Ran, F. A., Hsu, P. D., Wright, J., Agarwala, V., Scott, D. A., & Zhang, F. (2013). Genome engineering using the CRISPR-Cas9 system. *Nat Protoc, 8*(11), 2281-2308. doi:10.1038/nprot.2013.143

Ring, S., Weyer, S. W., Kilian, S. B., Waldron, E., Pietrzik, C. U., Filippov, M. A., . . . Muller, U. C. (2007). The secreted beta-amyloid precursor protein ectodomain APPs alpha is sufficient to rescue the anatomical, behavioral, and electrophysiological abnormalities of APP-deficient mice. *J Neurosci, 27*(29), 7817-7826. doi:10.1523/JNEUROSCI.1026-07.2007
